# Associations Between Dietary Patterns and the Occurrence of Hospitalization and Gastrointestinal Disorders—A Retrospective Study of COVID-19 Patients

**DOI:** 10.3390/nu17050800

**Published:** 2025-02-26

**Authors:** Viktoria Hawryłkowicz, Beata Stasiewicz, Sebastian Korus, Wiktoria Krauze, Kamila Rachubińska, Elżbieta Grochans, Ewa Stachowska

**Affiliations:** 1 Department of Human Nutrition and Metabolomics, Faculty of Health Sciences, Pomeranian Medical University in Szczecin, Broniewskiego 24, 71-460 Szczecin, Poland; viktoria.hawrylkowicz@pum.edu.pl (V.H.); 67392@student.pum.edu.pl (S.K.); wiktoria.krauze00@gmail.com (W.K.); 2 Department of Human Nutrition, The Faculty of Food Science, University of Warmia and Mazury in Olsztyn, Sloneczna 45f, 10-718 Olsztyn, Poland; 3 Department of Nursing, Faculty of Health Sciences, Pomeranian Medical University in Szczecin, 71-210 Szczecin, Poland; kamila.rachubinska@pum.edu.pl (K.R.); elzbieta.grochans@pum.edu.pl (E.G.)

**Keywords:** dietary patterns, nutrition, COVID-19, gastrointestinal disorders, hospitalization

## Abstract

During the COVID-19 pandemic, dietary habits in the population changed and sometimes deviated from healthy eating patterns, such as the Mediterranean diet. Based on reports on the quality of the diet of respondents to studies conducted at the beginning of the pandemic, it could be concluded that these new dietary habits are unfavorable for a good prognosis and the course of any disease and its severity of symptoms. This study decided to confront these assumptions with the results of people who had COVID-19. **Background/Objectives**: This study aimed to assess the associations between dietary patterns and the occurrence of hospitalization and gastrointestinal disorders among patients diagnosed with COVID-19. **Methods**: This study included 550 respondents who completed a survey up to 8 months after being diagnosed with COVID-19. The survey included 62 items from the FFQ-6^®^, GSRS, PAC-SYM and FACT-G7 standardized questionnaires. **Results**: Two dietary patterns (DPs) were identified: ‘Processed high fat/sugar/salt/meat/dairy/potatoes’ and ‘Semi-vegetarian’. Higher adherence to the ‘Processed’ DP was associated with higher odds of hospitalization due to COVID-19, a more severe course of the disease, and the highest intensity of gastrointestinal symptoms. Higher adherence to the ‘Semi-vegetarian’ DP was associated with lower odds of hospitalization due to COVID-19, a less severe course of the disease, and the lowest intensity of gastrointestinal symptoms. **Conclusions**: This study showed a strong harmful effect of high adherence to a processed dietary pattern on an increased incidence of hospitalization and gastrointestinal disorders among northwestern Polish adults during the COVID-19 pandemic, emphasizing the importance of a healthy diet.

## 1. Introduction

The coronavirus disease 2019 (COVID-19) pandemic has largely determined the health of people around the world, starting with its outbreak in 2019 in Wuhan, China. According to the latest data, there were 777,074,803 reported COVID-19 cases and over 7 million deaths [1]. COVID-19 is caused by severe acute respiratory syndrome coronavirus 2 (SARS-CoV-2) and predominant manifestations of the disease are respiratory disorders such as fever, sore throat, dry cough, runny nose and a general feeling of fatigue, and less frequently occurring symptoms are neurological and gastrointestinal (GI) disorders [2,3,4]. The most common GI symptoms caused by infection of SARS-CoV-2 are diarrhea, 32.5% [5]; nausea, 11.7% [5]; abdominal pain or discomfort, 4.4% [5]; and vomiting, 3.9% [6], occurring during the illness or its onset [5], but the presence of GI symptoms is not associated with the mortality of COVID-19 patients [7]. 

It is known so far that SARS-CoV-2 is able to bind to the angiotensin-converting enzyme 2 (ACE2) receptors, causing GI symptoms depending on the localization of these receptors in the digestive tract [8,9]. What is worth mentioning is that GI symptoms might also occur due to medications used for COVID-19, and these include abnormal appetite, diarrhea, nausea, vomiting, dry mouth, abdominal pain or discomfort, and constipation [10]. Licensed scales used to assess stomach and intestinal disorders are the IBS-VAS, GSRS, Gastroesophageal Reflux Disease Questionnaire (GerdQ), Post Infection IBS Scale (PI-IBS), Functional Bowel Disorders Severity Index (FBDSI), Irritable Bowel Syndrome-Quality of Life survey (IBS-QOL), Irritable Bowel Syndrome- Symptom Severity Scale (IBS-SSS), and Bristol Stool Form Scale (BSFS).

In the case of an infectious disease, the human body needs resources to fight the pathogen. This refers to avoiding possible nutritional deficiencies as they can determine the risk and severity of COVID-19 [11]. Nutritionally essential minerals and vitamins play an important role in the immune response. Among them can be mentioned trace elements, including zinc, selenium, iron, copper and magnesium; and vitamins such as D, E, A, B_12_, B_6_, C, and folate [12]. The immune function roles of these nutrients are (1) the maintenance of the structural and functional integrity of mucosal cells in innate barriers, for instance in the respiratory tract; (2) differentiation, proliferation, functioning, and movement of innate immune cells and T cells; (3) inflammation, antioxidant effects and role in oxidative bursts; (4) antibody production and development; (5) responses to antigens; and (6) the regulation of antimicrobial effects [12]. It was also shown that the omega-3 fatty acids eicosapentaenoic acid (EPA) and docosahexaenoic acid (DHA) are helpful for decreasing inflammation due to enzymatic oxygenation of specialized pro-resolving mediators (SPMs) known as resolvins, maresins, lipoxins and protectins following the initial stages of the inflammatory cascade in respiratory diseases [13,14,15]. Omega-3 polyunsaturated fatty acids supplementation was found to have promising effects on reducing CRP levels [16] and renal function and acidosis, resulting in improvement of clinical symptoms in COVID-19 [17]. The deficiencies of micronutrients and omega 3 acids might cause immunosuppression and increase the risk of infection. Nutritional deficiencies can also affect adaptive and innate immunity, which in turn affects the course and duration of infection. Moreover, mucosal-associated invariant T cells (MAIT), a type of innate-like T cell characterized by a semi-invariant T cell receptor, influence the polarization of adaptive lymphocyte responses and contribute to metabolic dysfunction [18]. Appropriate nutrient status seems to be crucial for preventing infections and reducing disease burden [19].

The proper level of nutritional content of the diet in terms of vitamins and microelements depends on the frequency and quantity of consumption of their rich sources, and therefore on dietary patterns. In this aspect, not only does the frequency of the supply of products rich in the above-mentioned components of the diet matter, but so does the simultaneous consumption of low amounts of processed products. Not to mention, the pro-inflammatory or anti-inflammatory potential of food plays a significant role in the immune response and inflammatory processes [20]. For instance, a high in saturated fats and trans fats diet might lead to higher pro-inflammatory TNFα, IFNγ, IL-6 and IL-1β cytokine concentrations [20]. The components of a Western-style diet, such as: refined carbohydrates increase levels of IL-6 and CRP and cause negative functioning of memory T cells; added sugars and fructose overconsumption are related to systemic pro-inflammatory status, cortisol hyperactivation and insulin resistance; fast foods decrease short chain fatty acid (SCFA) producers in the intestines and therefore cause disruption of the intestinal barrier by disrupting GALT immunity; excessive salt and meat consumption is associated with the metabolism of a compound called trimethylamine (TMA) by the gut microbiota, which is transformed into trimethylamine N-oxide (TMAO) in the liver and is associated with inflammatory pathways and cardiovascular disease risk [20]. On the other hand, the Mediterranean diet presents anti-inflammatory effects by reducing the level of interleukin (IL-6), high-sensitivity C-reactive protein (hs-CRP) and tumor necrosis factor (TNF-α) and might be the most recommended dietary pattern to follow [21]. Dietary patterns may be crucial in enhancing the immune responses against severe infections, leading to inflammation and increased cytokine secretion, and SARS-CoV-2 is being mentioned as an example [21].

A study from the beginning of the pandemic on the quality of diet during the lockdown emphasized that: (1) during the lockdown, respondents reported an increase in energy intake in the form of food and snacks; (2) about 20% of people surveyed practically never ate breakfast, which may lead to an increased risk of type 2 diabetes and heart disease; (3) almost one-third of respondents did not eat fresh vegetables and fruit every day; (4) about 37% of respondents ate sweets several times a week; and (5) the higher the BMI, the lower the consumption of vegetables and fruit [22]. Taking these reports into account, the authors of this paper decided to examine how the dietary patterns of respondents after COVID-19 affected their frequency of hospitalization due to disease.

Today, it is acknowledged that there is a clinically significant difference in the impact on health and immune function between two extremes in terms of their properties and components, namely the Mediterranean and Western diets [20]. Given the disturbing information that people with metabolic syndrome, overweight and obesity had poor dietary habits at the beginning of the pandemic [22], it is important to know how specific eating patterns influenced the severity of COVID-19 and its associated disorders. This study aimed to assess the associations between dietary patterns and the occurrence of hospitalization and gastrointestinal disorders among patients diagnosed with COVID-19.

## 2. Materials and Methods

### 2.1. Study Design and Sample Selection

**Figure 1:**
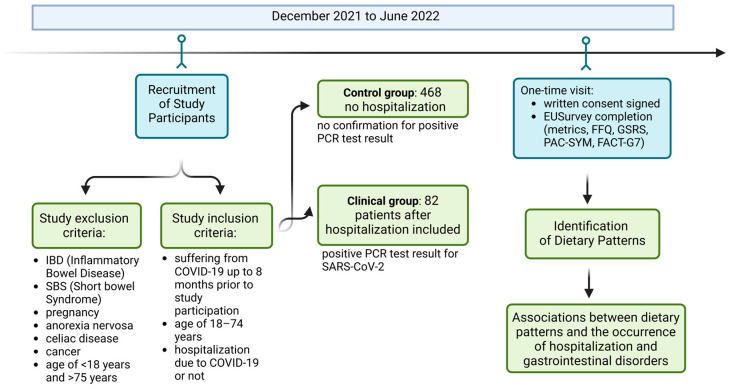
Study design work flow. Created in https://BioRender.com.

This study included 550 respondents who completed a survey up to 8 months after being diagnosed with COVID-19. The participants were 468 individuals without hospitalization (these patients suffered from COVID-19 at home, with no confirmation by a positive PCR test result) and 82 patients with a severe course of infection (positive PCR test result for SARS-CoV-2), aged 18–74 years old. Women constituted the majority of the respondents (89.8%). The average age of the respondents was 41.2 ± 11.4 years. Most respondents came from large cities with more than 200,000 inhabitants (40.6%) from northwestern Poland. Due to the SARS-CoV-2 virus pandemic and lockdown, data were collected online by an opensource surveying tool, EUSurvey [23], from December 2021 until June 2022. The study workflow is presented in Figure 1. The criteria for including patients in the clinical group were hospitalization due to severe COVID-19, confirmed by a test performed in the hospital, within the last 8 months before participation in the study; signing an informed consent form to participate in the study; age over 18 years; and no inflammatory bowel disease, cancer, short bowel syndrome, pregnancy, or anorexia nervosa. The criteria for including respondents in the control group were a diagnosis of SARS-CoV-2 infection within the last 8 months before participation in the study, age over 18 years, no inflammatory bowel disease, and no neoplastic disease. There were no requirements regarding the region of respondents, but the group of 82 people after hospitalization came from the city of Szczecin and its surrounding area, and the respondents completing the survey after recovering from COVID-19 at home came from all over the country.

### 2.2. Food Frequency Consumption and Dietary Patterns Identification

Dietary data were collected using a validated 62-item Food Frequency Questionnaire (62-item FFQ-6^®^) [24]. Respondents were asked about the frequency of consumption of 62 food groups in the last 12 months before participating in the study. Next, the frequency consumption was expressed as times/day after assigning the values as follows: ‘never or almost never’ = 0; ‘once a month or less’ = 0.025; ‘several times a month’ = 0.1; ‘several times a week’ = 0.571; ‘daily’ = 1; ‘several times a day’ = 2. Then, the frequency consumption of 62 food items (times/day) was aggregated into twenty-two food groups, standardized, and included in the Principal Component Analysis (PCA) with varimax rotation [25]. The full description of the twenty-two food groups aggregated is shown in Supplementary Table S1. Due to relatively low consumption and no impact on the value of explained variance, four food groups were excluded from the PCA: breakfast cereals, fruit/vegetable/vegetable–fruit juices, sweetened beverages and alcoholic drinks.

Two PCA-derived dietary patterns (DPs) were identified, called ‘Processed high fat, sugar, salt, meats, dairy, and potatoes’ and ‘Semi-vegetarian’. The plot of eigenvalues and the total variance were the main criteria for the identification of the PCA-derived DPs, and so the DPs were marked according to the main components with the absolute values of factor loadings >0.30 [25]. For further analysis, tercile intervals of each DP were calculated. The means of the frequency of food consumption (times/day) by dietary patterns (terciles) among the COVID-19 patients are provided in Supplementary Table S2. The details of the characteristics of the DPs are given in Section 3.

### 2.3. Gastric Scales: GSRS, PAC-SYM and FACT-G7

Outcome measures for the severity of gastrointestinal discomfort and symptoms included the Gastrointestinal Symptom Rating Scale (GSRS), The Patient Assessment of Constipation-Symptoms (PAC-SYM) and FACT-G7.

The Gastrointestinal Symptom Rating Scale (GSRS) is a self-report, 15-item questionnaire that measures the severity of a wide range of gastrointestinal symptoms, referring to diarrhea, constipation, bloating, abdominal pain, hunger pain, reflux, etc. Questions are rated on a 7-point Likert scale (1 = ‘No discomfort at all’, 2 = ‘Minor discomfort’, 3 = ‘Mild discomfort’, 4 = ‘Moderate discomfort’, 5 = ‘Moderately severe discomfort’, 6 = ‘Severe discomfort’ and 7 = ‘Very severe discomfort’) [26,27]. The GSRS score was calculated as the sum of all 15 responses and expressed in the range from 15 to 68 points. In terms of the intensity of discomfort (number of points) expressed on this scale, the respondents were divided into three terciles (bottom, middle and upper) of symptoms intensity for further analyses.

The Patient Assessment of Constipation-Symptoms (PAC-SYM) questionnaire is an important tool for the assessment of the severity of self-reported symptoms of patients suffering from chronic constipation [28,29]. This questionnaire contains 12 questions about the severity of symptoms such as painful abdominal cramps, painful bowel movements, urge to defecate, feeling of incomplete defecation, etc. Items are scored on a 5-point Likert scale (0 = ‘symptom absent’, 1 = ‘mild’, 2 = ‘moderate’, 3 = ‘severe’ and 4 = ‘very severe’). A mean total score in the range of 0–4 is generated by dividing the total score by the number of all questions; the lower the total score, the lower the symptom burden [29]. Analogous to the GSRS scores, the PAC-SYM scores were grouped into three terciles according to severity.

The FACT-G7 is a rapid version of the Functional Assessment of Cancer Therapy-General that can be used to assess top-rated symptoms and concerns for a broad spectrum of advanced cancers in clinical practice and research, such as self-reported feelings of lack of energy, pain, trouble sleeping, and dissatisfaction with the quality of the patient’s life [30,31]. Points from 1 to 5 were assigned according to the response to 7 questions (1 = ‘Not at all’, 2 = ‘A little bit’, 3 = ‘Somewhat’, 4 = ‘Quite a bit’, 5 = ‘Very much’). In this study, this scale was used to evaluate the subjective assessment of well-being after the disease and respondents’ health-related quality of life and links to dietary patterns.

Details of the GSRS, PAC-SYM and FACT-G7 scales and their components by the hospitalization status among COVID-19 patients are presented in Supplementary Table S3.

### 2.4. Statistical Analysis

Basic characteristics of the COVID-19 patients are expressed in means and standard deviations (SD). Differences in baseline sample characteristics were verified with a Kruskal–Wallis test (continuous data) or Pearson Chi^2^ test with Yates’ correction as necessary (categorical data) [25]. The sample percentage distributions of hospitalized COVID-19 patients were compared by tercile intervals of DPs. The associations of DPs with hospitalization risk or intensification of gastrointestinal disorders were assessed using logistic regression analysis. Then, the odds ratios (ORs) and 95% confidence interval (95% CI) of hospitalization or intensification of gastrointestinal disorders were calculated [25]. The reference categories (OR = 1.00) were the bottom terciles of each DP, the bottom terciles of gastric scales (GSRS, PAC-SYM and FACT-G7), and the lack of hospitalization. Two models were created: crude and adjusted for the potential confounders. The adjusted model of the ORs calculation of the hospitalization by the adherence to the DPs among COVID-19 patients included the following confounders: age (years), gender (men, women), place of residence, educational level, ever-smoking status (no, yes, occasionally), chronic diseases (no, yes), taking medication (no, yes), vitamin/mineral supplements use within the last 12 months (no, yes), and special diet or intake restrictions (no, yes). The adjusted model of the ORs calculation of the intensification of gastrointestinal disorders by the adherence to the DPs among COVID-19 patients included the same set of confounders and hospitalization (no/yes). The level of significance of the odds ratio was verified with a Wald’s test [25]. All statistical analyses were obtained using STATISTICA software (version 10.0 PL; StatSoft Inc., Tulsa, OK, USA; StatSoft, Krakow, Poland). A *p*-value < 0.05 was considered statistically significant.

## 3. Results

### 3.1. Baseline Sample Characteristics

Hospitalized patients with COVID-19 were older than non-hospitalized patients (54.5 vs. 38.8 years; *p* < 0.0001; Table 1). Significantly more hospitalized patients were 60 years or older (41.5 vs. 2.8%; *p* < 0.0001), had chronic diseases (70.4 vs. 42.3%; *p* < 0.0001), took medications (63.4 vs. 37.4%; *p* < 0.0001), were ever-smokers (50.0 vs. 30.3%; *p* = 0.0015), and did not eat regularly (29.3 vs. 15.8%; *p* = 0.0043). Significantly fewer hospitalized patients than non-hospitalized came from cities with more than 200,000 inhabitants (1.2 vs. 47.5%; *p* < 0.0001) and had a higher education level (36.3 vs. 84.0%; *p* < 0.0001; Table 1).

There were no significant associations in restriction in the consumption of sugar and sweets, fats and foods in the high fat content data, but there were some significant associations in restriction in the consumption of meat and meat products, raw vegetables, fruits and dairy products between the hospitalized and non-hospitalized groups of respondents. As the results show, hospitalized patients tended to manifest greater food reductions in the consumption of raw vegetables and lower reductions in the consumption of meat (Table 1).

### 3.2. Dietary Pattern Characteristics

Two main PCA-derived dietary patterns were identified: ‘Processed high fat, sugar, salt, meat, dairy, and potatoes’ (DP1) and ‘Semi-vegetarian’ (DP2). The total explained variance in the frequency of consumption of the 18 food groups was 30.9% (Table 2). The ‘Processed high fat, sugar, salt, meat, dairy, and potatoes’ DP was positively loaded by the frequency of consumption of refined grains, animal fats, processed meats, sugar/honey/sweets, cheese, potatoes, other fats (margarine, mayonnaise, dressings), sweetened milk drinks/flavored cheese, white meat, milk/fermented milk drinks/cheese curd and salty snacks (factor loadings from 0.30 to 0.66; Table 2). The ‘Semi-vegetarian’ DP was positively loaded by the frequency of consumption of vegetables, fruits, nuts/seeds, whole grains, legumes, milk/fermented milk drinks/cheese curd, eggs and fish (factor loadings from 0.31 to 0.67, Table 2). The sample characteristics by dietary patterns among the COVID-19 patients is shown in Supplementary Table S4.

### 3.3. Dietary Patterns and Hospitalization Among the COVID-19 Patients

Significantly more hospitalized than non-hospitalized COVID-19 patients were in the upper tercile of the ‘Processed high fat, sugar, salt, meat, dairy, and potatoes’ dietary pattern (54.9 vs. 29.5%; *p* < 0.0001; Table 3). Inversely, significantly fewer hospitalized patients were in the upper tercile of the ‘Semi-vegetarian’ pattern (18.3 vs. 35.9%; *p* = 0.0031). These results were confirmed in the logistic regression analysis, except for the adjusted model for the ‘Semi-vegetarian’ pattern (Table 4).

**Figure 2:**
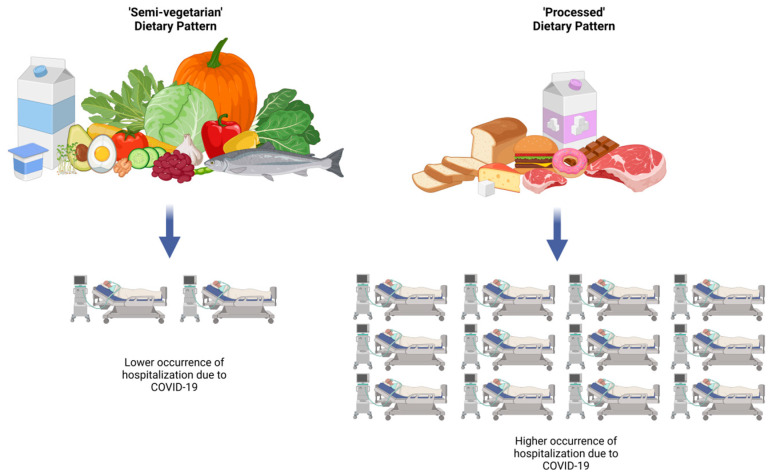
Associations between PCA-identified dietary patterns and hospitalization due to COVID-19. Created in https://BioRender.com.

The odds of hospitalization were more than four times higher among COVID-19 patients in the upper tercile of the ‘Processed high fat, sugar, salt, meat, dairy, and potatoes’ dietary pattern (OR crude = 4.29, 95% CI: 2.22–8.29, *p* < 0.001; OR adjusted = 4.40, 95% CI: 1.78–10.88, *p* < 0.01) than in the bottom tercile of this pattern (Table 4). A one-point increase in the score of the ‘Processed high fat, sugar, salt, meat, dairy, and potatoes’ DP was associated with an almost two times increased odds of hospitalization (OR crude = 1.78, 95% CI: 1.43–2.22, *p* < 0.001; OR adjusted = 1.93, 95% CI: 1.38–2.71, *p* < 0.01; Table 4). The odds of hospitalization among COVID-19 patients were lower by 66% in the upper tercile of the ‘Semi-vegetarian’ DP than in the bottom tercile of this pattern (OR crude = 0.34, 95% CI: 0.18–0.65, *p* < 0.001). A one-point increase in the score of the ‘Semi-vegetarian’ DP was associated with 33% decreased odds of hospitalization (OR crude = 0.67, 95% CI: 0.51–0.87, *p* < 0.01 (Table 4). These associations did not remain statistically significant in the adjusted models for the ‘Semi-vegetarian’ pattern. The summarized findings of this study regarding the associations between the PCA-identified dietary patterns and hospitalization among patients with COVID-19 are shown in Figure 2. Details of the frequency of single food group consumption by hospitalization among COVID-19 patients are presented in Supplementary Table S5.

### 3.4. Gastrointestinal Disorders and Dietary Patterns

In the upper tercile of the ‘Processed high fat, sugar, salt, meat, dairy, and potatoes’ dietary pattern were the COVID-19 patients with the highest GSRS scores, i.e., the most frequently reported gastrointestinal problems (*p* = 0.0011; Table 5). With the highest adherence to this pattern, these patients reported the highest intensity of digestive complaints on the GSRS scale (*p* = 0.0202), including heartburn (0.0020), hunger pains (*p* < 0.0001), rumbling (*p* = 0.0179), bloated stomach (*p* = 0.0277), breaking wind (*p* = 0.0109) and hard stools (*p* = 0.0367). Conversely, with increasing adherence to the ‘Semi-vegetarian’ pattern, the intensity of GSRS score symptoms decreased (*p* = 0.0064; *p* = 0.0152). The most notable results were reported in the reduction of constipation symptoms (*p* = 0.0294) and the urgent need to have a bowel movement in patients in the upper tercile of the ‘Semi-vegetarian’ DP (*p* = 0.0258; Table 5).

In the upper tercile of the ‘Processed high fat, sugar, salt, meat, dairy, and potatoes’ dietary pattern were also the COVID-19 patients with the highest intensity of single complaints on the PAC-SYM score, including discomfort in your abdomen (*p* = 0.0449), pain in your abdomen (*p* = 0.0235), stomach cramps (*p* = 0.0010), painful bowel movements (*p* = 0.0170) and rectal burning during or after a bowel movement (*p* = 0.0025; Table 5). In the upper tercile of the ‘Semi-vegetarian’ pattern, the problem of straining or squeezing to try to pass bowel movements was decreased (*p* = 0.0017).

The quality of life expressed by the FACT-G7 score was decreased among COVID-19 patients in the upper tercile of the ‘Processed high fat, sugar, salt, meat, dairy, and potatoes’ dietary pattern (*p* = 0.0001; Table 5). With the highest adherence to this pattern, the patients most frequently reported pain (<0.0001), trouble sleeping (*p* = 0.0002), not enjoying their lives (*p* = 0.0006) and not being content with the quality of life (*p* = 0.0078). Conversely, with increasing adherence to the ‘Semi-vegetarian’ pattern, the intensity of FACT-G7 score symptoms decreased (*p* = 0.0203). Patients seem to be more satisfied with their lives with greater adherence to the ‘Semi-vegetarian’ pattern (*p* = 0.0100; *p* = 0.0028; Table 5). The percentage distributions of single components of the GSRS, PAC-SYM and FACT-G7 scales by dietary patterns among COVID-19 patients are provided in Supplementary Table S6.

The logistic regression analysis showed that the odds of the highest intensity of gastrointestinal complaints expressed in the upper tercile of the GSRS score were more than two and three times higher among COVID-19 patients in the middle and upper tercile of the ‘Processed high fat, sugar, salt, meat, dairy, and potatoes’ dietary pattern, respectively (OR = 2.21, 95% CI: 1.28–3.83, *p* < 0.01; OR = 3.12, 95% CI: 1.74–5.63, *p* < 0.001) than in the bottom tercile of this pattern (Table 6). A one-point increase in the score of the ‘Processed high fat, sugar, salt, meat, dairy, and potatoes’ DP was associated with almost one and half times increased odds of the upper tercile of the GSRS score (OR = 1.47, 95% CI: 1.16–1.86, *p* = 0.01; Table 6). The odds of the upper tercile of the GSRS score among COVID-19 patients were lower by 43% and 69% in the middle and upper terciles of the ‘Semi-vegetarian’ DP than in the bottom tercile of this pattern (OR = 0.57, 95% CI: 0.33–0.97, *p* < 0.05; OR = 0.31, 95% CI: 0.17–0.56, *p* < 0.001). A one-point increase in the score of the ‘Semi-vegetarian’ DP was associated with 38% decreased odds of the highest intensity of gastrointestinal complaints (OR crude = 0.62, 95% CI: 0.49–0.79, *p* < 0.01; Table 6).

The odds of the highest intensity of constipation complaints expressed in the upper tercile of the PAC-SYM score were approximately three times higher among COVID-19 patients in the upper tercile of the ‘Processed high fat, sugar, salt, meat, dairy, and potatoes’ dietary pattern (OR = 3.11, 95% CI: 1.76–5.48, *p* < 0.001) than in the bottom tercile of this pattern (Table 7). A one-point increase in the score of the ‘Processed high fat, sugar, salt, meat, dairy, and potatoes’ DP was associated with one and half times increased odds of the upper tercile of the PAC-SYM score (OR = 1.50, 95% CI: 1.20–1.87, *p* < 0.001; Table 7). The odds of the upper tercile of the PAC-SYM score among COVID-19 patients were lower by 48% and 65% in the middle and upper terciles of the ‘Semi-vegetarian’ DP than in the bottom tercile of this pattern (OR = 0.52, 95% CI: 0.31–0.88, *p* < 0.05; OR = 0.35, 95% CI: 0.20–0.60, *p* < 0.001). A one-point increase in the score of the ‘Semi-vegetarian’ DP was associated with 36% decreased odds of the highest intensity of gastrointestinal complaints (OR crude = 0.64, 95% CI: 0.51–0.81, *p* < 0.01; Table 7).

**Figure 3:**
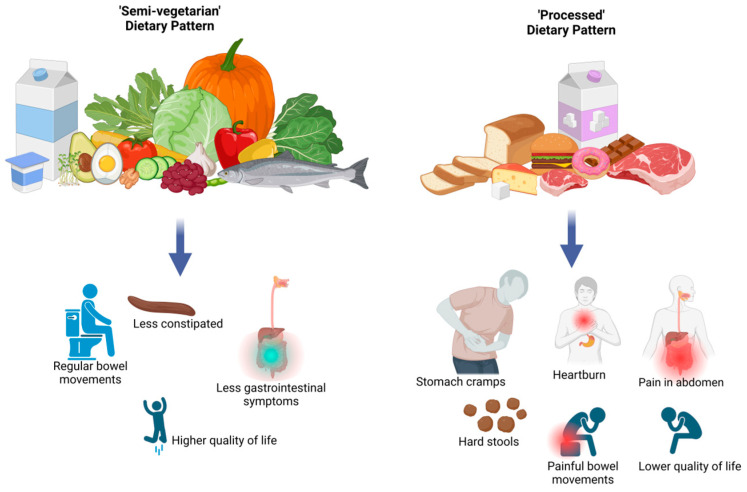
Associations between PCA-identified dietary patterns and the severity of reported gastrointestinal disorders among patients with COVID-19. Created in https://BioRender.com.

The odds of the highest intensity of decreased quality of life expressed in the upper tercile of the FACT-G7 score were more than two and four times higher among COVID-19 patients in the middle and upper tercile of the ‘Processed high fat, sugar, salt, meat, dairy, and potatoes’ dietary pattern, respectively (OR = 2.22, 95% CI: 1.27–3.89, *p* = 0.01; OR = 4.55, 95% CI: 2.43–8.50, *p* < 0.001) than in the bottom tercile of this pattern (Table 8). A one-point increase in the score of the ‘Processed high fat, sugar, salt, meat, dairy, and potatoes’ DP was associated with more than one and half times increased odds of the upper tercile of FACT-G7 score (OR = 1.54, 95% CI: 1.21–1.97, *p* < 0.001; Table 8). The odds of the upper tercile of FACT-G7 score among COVID-19 patients were lower by 55% in the upper terciles of the ‘Semi-vegetarian’ DP than in the bottom tercile of this pattern (OR = 0.45, 95% CI: 0.25–0.78, *p* = 0.01). A one-point increase in the score of the ‘Semi-vegetarian’ DP was associated with 30% decreased odds of the highest problem with the self-assessed quality of life (OR = 0.70, 95% CI: 0.55–0.89, *p* = 0.01 (Table 8). The summarized findings of the current study regarding the associations between the PCA-identified dietary patterns and the severity of reported gastrointestinal disorders among patients with COVID-19 are shown in Figure 3.

## 4. Discussion

To the best of the authors’ knowledge, this is the first study to comprehensively evaluate the association between dietary patterns and the occurrence of hospitalization and gastrointestinal disorders among patients diagnosed with COVID-19. The obtained results showed that the adherence to the ‘Processed high fat, sugar, salt, meat, dairy, and potatoes’ pattern was associated with an increase in the intensity of gastrointestinal complaints expressed in all three gastric scales: GSRS, PAC-SYM and FACT-G7, as well as with an increase the odds of hospitalization due to COVID-19. Inversely, adherence to the ‘Semi-vegetarian’ pattern decreased the intensity of gastrointestinal complaints. In regards to hospitalization, an inverse association with the ‘Semi-vegetarian’ pattern was found, but it disappeared after adjustment.

### 4.1. Dietary Patterns and Severity of COVID-19

The obtained findings show that high adherence to the ‘Processed high fat, sugar, salt, meat, dairy, and potatoes’ DP increased the occurrence of hospitalization due to COVID-19 by more than four-times. This negative effect could result from elevated consumption of high-processed foods which are ‘empty calories’ high in sugar and animal fat content, related to positive energy balance and obesity [32]. The evidence showed that a higher BMI is associated with an increased severity of COVID-19 and the possibility of hospitalization [33]. The so-called “Western diet”, composed mainly of highly processed foods, is described as poor in the quality of micronutrient content [34]. A diet of mostly processed foods is associated with low consumption of vegetables, legumes, and whole grains, which are sources of essential vitamins, minerals, and nutrients [35]. Highly processed dietary patterns may result in intestinal barrier dysfunction and increased intestinal permeability, which may be related to inflammation [36,37]. Moreover, these dietary patterns increase oxidative stress by high reactive oxygen species (ROS) production and then lead to decreased immunity, and increase the severity of viral infection. To fight this imbalance, obtaining natural antioxidants from food is crucial [32]. In the available studies conducted during the COVID-19 pandemic, the PCA-dietary patterns were not identified; therefore, it is not possible to directly compare them with the results obtained in the current study. However, many studies were focused on evaluating the dietary habits of different populations during and after the COVID-19 pandemic, and a significant increase in the consumption of ultra-processed foods including sweets, salty snacks, fast foods, soft drinks, and meat products was observed [22,33,38,39]. This was explained by the lockdown and the associated longer time spent in front of the TV or computer screen and increased stress [33]. These lifestyle changes may have long-term effects on health [39]. To prevent this, it is necessary to avoid or limit the consumption of highly processed foods that are high in sugar, salt, saturated and trans fatty acids, cholesterol, and food additives. It is not possible to eliminate all processed foods from our diet. However, there are some suggestions for the general public to decrease the consumption of highly processed foods that have been proposed. First of all, if you want some sweets, eat fruit, and if you want salty snacks, eat nuts or seeds, but without salt. Instead of salt, use different herbs and spices. Instead of a sweetened drink, add a slice of citrus to water and make lemonade. Whenever you are at home, cook, preferably with loved ones; it is a good way to spend time together and build relationships.

Conversely to the ‘Processed’ DP, high adherence to the ‘Semi-vegetarian’ DP decreased the occurrence of hospitalization among COVID-19 patients by 66%. This beneficial effect of the ‘Semi-vegetarian’ pattern disappeared after adjustment. This could be due to the presence of other lifestyle factors, such as smoking, which neutralized the beneficial features of the diet. The ‘Semi-vegetarian’ DP was characterized by a relatively high frequency of consumption of vegetables, fruits, nuts/seeds, whole grains, legumes, milk/fermented milk drinks/cheese curd, eggs and fish. These features are similar to the Mediterranean diet, which has been described as one of the healthiest diets in the world [40]. Similar to the present results, a recent systematic review and meta-analysis provided evidence that high-quality plant-based dietary patterns decreased the risk of hospitalization due to SARS-CoV-2 infection by 62% [41]. Findings from single studies, including the prospective SUN Project, also indicated that better adherence to healthy dietary patterns like the Mediterranean diet was associated with a lower COVID-19 risk and severity by 64% and 77%, respectively [42,43]. This protective effect can be explained by several plausible mechanisms. The main determinant of the course of infectious disease is the immune response [44]. An appropriate immune response promotes a mild course of infection, while the opposite situation leads to a cytokine storm and a severe course of the disease. This is promoted by inflammation and oxidative stress. Due to their composition, diets like Semi-vegetarian or Mediterranean provide essential vitamins, minerals, polyphenols, and phytochemicals, which are natural antioxidants and are needed to counteract oxidative damage and maintain an active immune response, as well as polyunsaturated fatty acids (omega-3) with anti-inflammatory effects [32,40,44]. There is growing evidence suggesting that the intake of antioxidants, including selenium, zinc, and vitamins D and E, above the currently recommended levels, can improve the immune system and enhance resistance to infection [32]. The less frequent hospitalizations in COVID-19 patients may have resulted from the antioxidant and anti-inflammatory properties of these balanced dietary patterns [37]. The beneficial role of these components in reducing the risk of infection or having a mild course of COVID-19 was confirmed by the results of studies in which their concentration in the blood was assessed. Many studies have shown that low blood levels of nutrients, including vitamin D, vitamin B12, selenium, zinc, calcium, and magnesium, were significantly correlated with an increased incidence of SARS-CoV-2 infection, severe forms of COVID-19, and its negative clinical outcomes, including intensive care support of the patients, and worse survival of COVID-19 [44,45,46,47,48,49]. These findings suggest that the mentioned nutrients have a potential role as biomarkers of COVID-19 progression, and their deficiencies indicate a poor prognosis in patients with COVID-19 [49].

### 4.2. Dietary Patterns and Gastrointestinal Disorders

In this study, it has been shown that gastroenterological disorders predominated in the group of people with an upper tercile of the processed pattern. Perhaps this is related to the salty snacks’ intake, low physical activity in lockdown or unhealthy dietary choices such as sugary snacks, honey, sweetened milk drinks and yogurts and salty snacks. What is crucial to mention is that manifestation of GI disorders was conditioned by the location of ACE2 receptors used by SARS-CoV-2 to enter the cells in the gastrointestinal tract, which independently caused digestive disorders in some infected patients [8,9]. There are not many studies that have used the Gastrointestinal Symptom Rating Scale (GSRS), PAC-SYM and FACT-G7 in relation to COVID-19 demonstrating the influence of diet quality on the score on these scales. There are research results available that discuss the low FODMAP diet or probiotic therapy, which are beyond the scope of this work.

In this study, a high dietary processing level was associated with heartburn. Participants who had the highest processed diet intake were more likely to report heartburn symptoms but not reflux symptoms. Heartburn is an unpleasant burning sensation in the esophagus that occurs as a result of stomach contents flowing back into the esophagus (gastroesophageal reflux). Foods and eating habits that increase the risk of heartburn include frequent consumption of fatty foods, fried foods, fast food or fatty meats, chocolate, alcohol or carbonated drinks, which can lead to a weakening of the lower esophageal sphincter, which facilitates the reflux of stomach acids [50,51,52]. Due to the difficult digestion of fatty foods, the time it takes for the stomach to digest the contents is longer, which additionally increases the risk of heartburn. In this study, the infection itself was a factor in the occurrence of heartburn and respondents with the DP1 dietary pattern showed more frequent consumption of processed meals, possibly worsening the sensation of this gastrointestinal disorder. In 2019, a study by Kamiński et al. [53] was published, which pointed out that searching for information on the Internet on heartburn was high in developed countries, where the Western diet prevails as dietary pattern. Interestingly, the study showed that in some countries such as Canada, Germany, Poland, and the United Kingdom, query numbers were significantly lower in summer but higher in colder seasons. This observation could be explained by seasonal variations in fat dietary intake since there is less access to fresh seasonal vegetables and fruits in these countries in autumn and winter than in summer [53]. The presence of fatty foods in the duodenum can stimulate a release of cholecystokinin, which reduces the pressure of the lower esophageal sphincter, leading to gastroesophageal reflux [54]. In another study conducted through an online survey of 1146 participants, it was shown that the prevalence of GERD was higher in non-vegans (11%) than in vegans (6%) [55]. These results confirm other reports that vegetarian diets and no intake of meat were negatively related to gastroesophageal reflux disease (GERD), while meat (daily meat, fish, and egg intake) and fat (high-fat diet) consumption were positively related to GERD [56].

Bloating is another GI problem due to worse tolerance of dietary fiber and it can be related to a disadvantageous microbiota composition or changes, long-term constipation or lower physical activity, that are common in times of high technical development or lockdown related to the COVID-19 pandemic. As bloating and breaking wind/gases can be a normal response to drastic changes in diet, such as a rapid increase in dietary fiber intake, it is caused by low consumption of this ingredient over a specific period of time. In an observational study conducted by Mitsou et al., it was shown that adherence to the Mediterranean Diet (MD) is associated with a decrease in bloating and increase in fecal moisture and defecation frequency [57]. Individuals in the high compliance to MD tercile reported a greater total number of bowel movements over the seven-day period compared to the low-adherence tercile but no differences in the Bristol stool scale were detected. What is more, scores of pain, bloating and the sum of gastrointestinal symptoms were higher in the high-adherence tercile compared with the low-adherence tercile [57]. Similar results were provided by the study by Barber et al., which compared the Mediterranean and Western diets [58]. The MD was associated with significantly higher scores of rumblings, also known as borborygmi, and sensations of flatulence than the Western diet. The MD was associated with more stool output and a softer stool consistency compared to the Western diet, but no differences in the number of daily bowel movements were observed [58]. This may be explained by the influence of abrupt changes in nutrition, more precisely the consumption of a high-fiber MD diet, on the increased fermentation process of the microbiota during the production of bacterial metabolites, which manifests itself as flatulence in the first weeks after the intervention.

Constipation is defined as difficulties in passing the stool, fewer bowel movements and a hard, compact consistency of the stool that can be measured by Bristol stool scale. Functional constipation is characterized by repetitive difficult, incomplete, or infrequent defecation, and affects approximately 10.1% (Rome IV criteria) of the worldwide population of adults, resulting in considerable reductions in quality of life [59,60,61]. The most common dietary factor associated with constipation is insufficient amount of soluble and insoluble fiber in daily nutrition. Soluble fiber (found in fruit, flax seeds, and oats) binds water, which helps create a soft consistency of the stool. Insoluble fiber (found in whole grains, vegetables, and seeds) accelerates food passage through the intestines, preventing the stool from dehydrating. Four weeks of supplementation with fiber (polydextrose, oligosaccharides, psyllium husk, or wheat bran) or increasing its intake in consistently constipated individuals resulted in a significant increase in bowel movements frequency but not in the placebo group [62]. In another study it was found that consumption of green kiwifruit for four weeks was associated with a clinically relevant increase in complete spontaneous bowel movements per week and significantly improved measures of gastrointestinal comfort in constipated participants [63]. Participants of this study showing an upper tercile in the DP1 were characterized by lower consumption of fiber-rich products of both factions, which positively correlated with increased intensification of the reported hard stools or painful bowel movements. This observation confirms the role of a diet poor in sources of food fiber in deterioration of the regularity of defecation.

### 4.3. Dietary Factors Contributing to Intestine Wall Maintenance

Gut health is a key element of overall well-being. In recent years, more and more research has confirmed that nutrition has a direct impact on the functioning of the digestive system, and in particular the intestines and other organs [63,64,65,66,67,68,69]. Quality of nutrition directly affects the condition of gut function. A diet rich in whole grains, vegetables, fruits and fermented products supports digestion and indirectly protects against pathogens. This is mainly due to SCFAs production by the microbial fermentation of prebiotics, such as dietary fiber [70]. As SCFAs production depends on modulation by the diet microbiome, it is obvious that nutrition has the potential to regulate the immune system. A change in diet is a decisive stimulus provoking quick changes in the composition of the microbiota, even within 24 h [71]. However, it is important that permanent, clinically significant changes occur with long-term habits and repeated activities that in summary create dietary patterns [58]. The dietary choices of the respondents of this study could be dictated by social distance, lockdown, remote work, stress, reduced physical activity and finally more time spent on snack consumption [72,73,74]. Despite the fact that the pandemic has ended, some people continue to live in a similar way: they present negligible physical activity and adverse dietary choices and the result is overweight or obesity.

One of the most important elements of the diet that affect intestinal transit and immune system functions is the form and amount of daily dietary fiber. It comes in two main fractions: soluble and insoluble. Soluble fiber, present in fruits, vegetables, and legumes, among others, creates a gel in the intestines that regulates bowel movements. Insoluble fiber, found in whole grains, speeds up the passage of food through the intestines, which helps prevent constipation. SCFAs such as acetate, butyrate and propionate are metabolites produced from fiber and they strengthen the mucosal barrier via its trophic properties for intestinal epithelial cells [75]. A lower abundance of SCFAs producers may be related to the ‘leaky gut’ hypothesis due to increased serum zonulin and upregulated pro-inflammatory cytokines (TNF-α, HMGB-1, IL-6, IL1-ra, and MIP-1β) [75]. As an example, for a diet rich in diverse nutrients and micronutrients, and fiber of both fractions, the recommendations of the Mediterranean diet may be considered the gold standard.

### 4.4. Strengths and Limitations

This study comprehensively presents associations between dietary patterns and the occurrence of hospitalization and gastrointestinal disorders among patients diagnosed with COVID-19. Three tools, GSRS, PAC-SYM and FACT-G7, were taken into account in assessing the severity of gastrointestinal disorders. Another strength of this study is that the respondents’ diets were assessed by PCA-derived dietary patterns, not based on a single food component intake. The dietary pattern identification approach is the currently recommended approach for disease associations [25]. In dietary data collection, a validated interviewer-administered FFQ was used [24]. Finally, in the adjusted model of the occurrence of hospitalization and gastrointestinal disorders, a wide range of potential confounders, including socioeconomic, lifestyle, and medical factors, were also taken into account.

This study also had some limitations related to its retrospective design. Compared to prospective studies, we could not assess the effect of diet on the risk of hospitalization and the severity of gastrointestinal disorders among patients with COVID-19. Only the assessment of the associations between these variables was possible [76,77]. Next, dietary data were obtained based on the food frequency consumption during the last twelve months before participation in this study. These data may be burdened by respondents’ memory bias [76]. In addition, respondents tend to overestimate the consumption of pro-healthy foods such as vegetables or fruits and underestimate the consumption of foods known as unhealthy such as sweets or fast foods. Another limitation of the study was a lack of measurements of blood concentration of nutrients, which are biomarkers of nutrient intake from all sources, including diet and supplements, and endogenous synthesis, as well as not influenced by a recall bias [76,77]. However, the results of blood tests during the disease could be influenced by the severity of disease, and then lead to reverse causation. Therefore, the measurements of the blood concentration of nutrients would be justified in prospective studies, at the beginning of the COVID-19 diagnosis and during treatment [47].

### 4.5. Clinical Impact and Future Directions

The COVID-19 pandemic, caused by SARS-CoV-2, has highlighted the potential role of nutrition in modifying disease severity and outcomes. This is confirmed by the results of our findings, where patients with COVID-19 whose diet was based on processed foods were more likely to require hospitalization and reported gastrointestinal complaints. Therefore, the present findings indicate the need for education and the implementation of healthy dietary principles in the prevention of both diseases, non-communicable and communicable. This approach could be an effective strategy to reduce the risk of severe diseases by implementing simple dietary principles at the population level. Furthermore, to better understand the role of nutrients and the mechanisms of their influence on the risk of infection and its course, as well as other health implications, further randomized clinical trials are needed among patients with infectious diseases, where, in addition to dietary data, blood levels of nutrients will be monitored and taken into account in the treatment protocol.

## 5. Conclusions

The present study provides interesting insight into the importance of diet on the severity of infectious diseases, for example COVID-19, and reported gastrointestinal disorders. The obtained findings have shown strong harmful effects of high adherence to a processed, high fat, sugar, salt, meat, dairy, and potatoes dietary pattern, with an increased incidence of hospitalization and gastrointestinal disorders among northwestern Polish adults during the COVID-19 pandemic. On the other hand, there was a strong beneficial effect of high adherence to a balanced semi-vegetarian pattern regarding a decreased incidence of gastrointestinal disorders and a slight beneficial effect on reduced hospitalization due to COVID-19.

The obtained results confirm that nutrition is not only important in the context of preventing non-communicable diseases, but may be also important in the prevention of infectious diseases in an indirect way. Prevention of infectious diseases will depend on the efficiency of the immune system, and thus the nutritional status, which ultimately depends on the quality of the diet. Based on the findings obtained, to strengthen the immune system for the prevention of infectious diseases, it is recommended to primarily limit the consumption of highly processed foods and increase the quality of the diet by choosing mainly plant-based diets with added fish and a limited amount of meat. These dietary models are called Semi-vegetarian or Mediterranean, and they contain all nutrients, which makes them balanced and valuable.

## Figures and Tables

**Table 1:** Basic characteristics of the COVID-19 patient sample (% or mean ± SD).

Variables	Total Sample	Hospitalization		*p*-Value
		No	Yes	
Sample size (n)	550	468	82	
Gender				
men	10.2	4.5	42.7	<0.0001
women	89.8	95.5	57.3	
Age (years ^#^)	41.2 ± 11.4	38.8 ± 9.4	54.5 ±12.8	<0.0001
18.0–29.9	14.2	16.5	1.2	
30.0–39.9	34.9	39.1	11.0	
40.0–49.9	31.8	33.1	24.4	<0.0001
50.0–59.9	10.5	8.5	22.0	
≥60.0	8.5	2.8	41.5	
Place of residence				
village	14.3	14.5	13.4	
town < 50,000 inhabitants	16.7	16.8	15.9	<0.0001
city (50,000–200,000 inhabitants)	28.4	21.2	69.5	
city (>200,000 inhabitants)	40.6	47.5	1.2	
Educational level				
primary	1.1	0.2	6.3	
basic vocational	3.3	1.5	13.8	<0.0001
secondary	18.6	14.3	43.8	
higher	77.0	84.0	36.3	
Chronic diseases	46.4	42.3	70.4	<0.0001
Taking medication	41.3	37.4	63.4	<0.0001
Ever smoker				
no	48.5	50.0	40.0	
yes	33.2	30.3	50.0	0.0015
occasionally	18.3	19.7	10.0	
Current smoker	8.2	8.8	4.9	0.2432
Vitamin/mineral supplements use	90.4	92.3	79.3	<0.0001
vitamin supplements	43.4	42.3	50.0	0.1931
folic acids supplements	36.2	37.4	29.6	0.1796
iron supplements	32.1	33.9	22.5	0.0452
zinc supplements	49.7	51.8	37.7	0.0221
vitamin C supplements	64.0	64.7	59.5	0.3698
Dietary habits				
Number of meals				
1–2	4.0	4.2	2.4	
3	33.5	33.1	35.4	0.7326
4	50.0	50.6	46.3	
≥5	12.5	12.0	15.9	
Regular meal times				
no	17.8	15.8	29.3	
rather yes	60.9	61.3	58.5	0.0043
definitely yes	21.3	22.9	12.2	
Special diet or intake restrictions	58.5	60.7	46.3	0.0150
Overall decrease in food consumption	38.5	38.9	36.6	0.6926

Self-declared use of vitamin and/or mineral supplements within the last 12 months; %—sample percentage; ^#^ mean and standard deviation (SD); *p*-value—level of significance verified with chi^2^ test (categorical variables) or Kruskal–Wallis’ test (continuous variables); *p* < 0.05—statistically significant.

**Table 2:** Factor loadings for food groups in Principal Component Analysis (PCA)-derived dietary patterns among COVID-19 patients (*n* = 550).

Food Groups	PCA-Derived Dietary Patterns	
	‘Processed High Fat, Sugar, Salt, Meat, Dairy, and Potatoes’	‘Semi-Vegetarian’
Refined grains	**0.66**	0.03
Animal fats	**0.62**	0.01
Processed meats	**0.62**	−0.07
Sugar, honey and sweets	**0.60**	−0.18
Cheese	**0.59**	0.12
Potatoes	**0.47**	0.25
Other fats (margarine, mayonnaise, dressings)	**0.46**	−0.04
Sweetened milk drinks and flavored cheese	**0.34**	0.07
White meat	**0.33**	0.27
Milk, fermented milk drinks and cheese curd	**0.33**	**0.48**
Salty snacks	**0.30**	−0.17
Vegetables	−0.13	**0.67**
Fruits	−0.02	**0.64**
Nuts and seeds	−0.25	**0.62**
Whole grains	0.05	**0.60**
Legumes	−0.06	**0.57**
Eggs	0.17	**0.45**
Fish	0.04	**0.31**
Share in explaining the variance (%)	16.2	14.7

Bolded values are marked for the main components of the PCA-derived dietary patterns with absolute loadings ≥ 0.3.

**Table 3:** PCA-derived dietary patterns in association with hospitalization among COVID-19 patients (%).

Variables	Total Sample	Hospitalization		*p*-Value
		No	Yes	
Sample size	550	468	82	
PCA-derived dietary patterns				
‘Processed high fat, sugar, salt, meat, dairy, and potatoes’				
terciles				
bottom	33.5	36.5	15.9	
middle	33.3	34.0	29.3	<0.0001
upper	33.3	29.5	54.9	
‘Semi-vegetarian’				
terciles				
bottom	33.3	31.0	46.3	
middle	33.5	33.1	35.4	0.0031
upper	33.3	35.9	18.3	

%—sample percentage; *p*-value—level of significance assessed by chi^2^ test; *p* < 0.05.

**Table 4:** Odds ratios (ORs (95% CI)) of the hospitalization by the adherence to the dietary patterns among COVID-19 patients (*n* = 550).

Dietary Patterns	Terciles	Hospitalization			
		Yes (Ref. No)			
		OR_crude_	95% CI	OR_adj_	95% CI
‘Processed high fat, sugar, salt, meat, dairy, and potatoes’	bottom (ref.)	1.00		1.00	
	middle	1.99	0.98; 4.04	1.90	0.65; 5.61
	upper	4.29 ***	2.22; 8.29	4.40 **	1.78; 10.88
	score (1-point increase)	1.78 ***	1.43; 2.22	1.93 **	1.38; 2.71
‘Semi-vegetarian’	bottom (ref.)	1.00		1.00	
	middle	0.71	0.42; 1.22	0.64	0.30; 1.39
	upper	0.34 ***	0.18; 0.65	0.57	0.22; 1.44
	score (1-point increase)	0.67 **	0.51; 0.87	0.85	0.57; 1.27

ref.—reference category; OR_crude_—crude model; OR_adj_—model adjusted for age (years), gender (men, women), place of residence, educational level, ever smoking status (no, yes, occasionally), chronic diseases (no, yes), taking medication (no, yes), vitamin/mineral supplements use within the last 12 months (no, yes), and special diet or intake restrictions (no, yes); 95% CI—95% confidence interval; *p*-value—the level of significance was assessed by Wald’s test; ** *p* < 0.01, *** *p* < 0.001.

**Table 5:** The selected gastric scales by dietary patterns among the COVID-19 patients (%) or mean (SD).

Variable	Total Sample	Dietary Patterns (Terciles/Levels)							
		‘Processed High Fat, Sugar, Salt, Meat, Dairy, and Potatoes’				‘Semi-Vegetarian’			
		Bottom	Middle	Upper	*p*-Value	Bottom	Middle	**Upper**	***p*-Value**
Sample size (n)	550	184	183	183		183	184	183	
GSRS_sum of points ^#^	30.1 ± 11.7	27.4 ± 9.8	31.1 ± 11.9	32.0 ± 12.8	0.0011	31.9 ± 12.0	30.4 ± 12.2	28.1 ± 10.7	0.0064
terciles									
bottom	37.3	44.0	33.3	34.4		31.7	38.6	41.5	
middle	28.7	31.0	30.1	25.1	0.0202	26.2	26.6	33.3	0.0152
upper	34.0	25.0	36.6	40.4		42.1	34.8	25.1	
GSRS_Components (points) ^#^									
pain or discomfort in your upper abdomen or the pit of your stomach	2.3 ± 1.4	2.2 ± 1.3	2.4 ± 1.5	2.4 ± 1.5	0.2788	2.5 ± 1.5	2.3 ± 1.5	2.2 ± 1.2	0.0916
heartburn	1.5 ± 1.1	1.4 ± 1.0	1.4 ± 0.9	1.7 ± 1.3	0.0020	1.6 ± 1.1	1.6 ± 1.1	1.4 ± 0.9	0.5689
acid reflux	1.6 ± 1.1	1.5 ± 1.0	1.5 ± 1.0	1.7 ± 1.3	0.2272	1.7 ± 1.3	1.6 ± 1.1	1.5 ± 0.9	0.6380
hunger pains	2.0 ± 1.2	1.7 ± 1.0	2.0 ± 1.2	2.4 ± 1.4	<0.0001	2.2 ± 1.3	2.0 ± 1.2	1.9 ± 1.1	0.0294
nausea	1.5 ± 1.1	1.5 ± 1.0	1.6 ± 1.1	1.6 ± 1.2	0.6144	1.6 ± 1.2	1.5 ± 1.1	1.4 ± 1.0	0.3054
rumbling	2.2 ± 1.3	2.0 ± 1.1	2.3 ± 1.3	2.3 ± 1.3	0.0179	2.3 ± 1.3	2.3 ± 1.3	2.1 ± 1.1	0.3691
bloated stomach	2.7 ± 1.6	2.4 ± 1.4	2.9 ± 1.6	2.7 ± 1.7	0.0277	2.9 ± 1.6	2.6 ± 1.6	2.5 ± 1.4	0.0588
breaking wind	3.0 ± 1.5	2.7 ± 1.3	3.1 ± 1.6	3.1 ± 1.5	0.0109	3.2 ± 1.5	3.0 ± 1.6	2.8 ± 1.4	0.0691
constipation	1.9 ± 1.5	1.8 ± 1.4	2.0 ± 1.5	2.0 ± 1.5	0.0653	2.1 ± 1.6	2.0 ± 1.5	1.7 ± 1.3	0.0294
diarrhoea	1.5 ± 1.1	1.4 ± 0.9	1.6 ± 1.2	1.5 ± 1.1	0.2012	1.5 ± 1.1	1.5 ± 1.1	1.4 ± 0.9	0.4283
hard stools	2.0 ± 1.4	1.7 ± 1.2	2.1 ± 1.5	2.2 ± 1.6	0.0367	2.2 ± 1.6	2.0 ± 1.4	1.8 ± 1.3	0.0415
loose stools	1.7 ± 1.2	1.6 ± 1.0	1.8 ± 1.3	1.8 ± 1.2	0.0969	1.7 ± 1.2	1.7 ± 1.2	1.7 ± 1.1	0.9703
urgent need to have a bowel movement	1.6 ± 1.1	1.5 ± 1.0	1.7 ± 1.2	1.7 ± 1.2	0.1063	1.6 ± 1.1	1.7 ± 1.2	1.5 ± 1.0	0.0258
sensation of not completely emptying the bowels	2.0 ± 1.4	1.8 ± 1.3	2.0 ± 1.4	2.1 ± 1.4	0.1851	2.1 ± 1.5	1.9 ± 1.3	1.9 ± 1.4	0.3391
belching	2.6 ± 1.6	2.4 ± 1.5	2.7 ± 1.6	2.7 ± 1.6	0.1033	2.8 ± 1.7	2.5 ± 1.6	2.4 ± 1.5	0.2777
PAC-SYM_sum of points ^#^	7.5 ± 7.8	6.2 ± 7.0	7.6 ± 7.5	8.8 ± 8.5	0.0140	8.6 ± 8.0	7.3 ± 7.5	6.6 ± 7.6	0.0085
terciles									
bottom	42.5	48.4	42.6	36.6		33.3	42.9	51.4	
middle	23.8	26.6	23.0	21.9	0.0220	25.7	25.0	20.8	0.0114
upper	33.6	25.0	34.4	41.5		41.0	32.1	27.9	
PAC-SYM_Components (points) ^#^									
discomfort in your abdomen	0.8 ± 0.9	0.6 ± 0.8	0.8 ± 1.0	0.8 ± 1.0	0.0449	0.9 ± 1.0	0.7 ± 1.0	0.7 ± 0.8	0.0578
pain in your abdomen	0.5 ± 0.8	0.4 ± 0.7	0.5 ± 0.9	0.7 ± 0.9	0.0235	0.6 ± 0.9	0.5 ± 0.8	0.5 ± 0.8	0.1713
bloating in your abdomen	1.2 ± 1.1	1.0 ± 1.0	1.3 ± 1.1	1.2 ± 1.1	0.1124	1.3 ± 1.1	1.1 ± 1.0	1.1 ± 1.0	0.2734
stomach cramps	0.5 ± 0.8	0.3 ± 0.7	0.5 ± 0.8	0.6 ± 0.9	0.0010	0.5 ± 0.8	0.5 ± 0.8	0.4 ± 0.8	0.2521
painful bowel movements	0.4 ± 0.9	0.3 ± 0.8	0.4 ± 0.9	0.6 ± 1.0	0.0170	0.6 ± 1.0	0.4 ± 0.8	0.3 ± 0.8	0.0047
rectal burning during or after a bowel movement	0.5 ± 0.9	0.4 ± 0.7	0.4 ± 0.8	0.7 ± 1.1	0.0025	0.5 ± 0.8	0.5 ± 0.9	0.5 ± 0.9	0.2021
rectal bleeding during or after a bowel movement	0.3 ± 0.8	0.3 ± 0.7	0.3 ± 0.7	0.4 ± 0.9	0.1541	0.4 ± 0.9	0.3 ± 0.7	0.3 ± 0.8	0.0660
incomplete bowel movement, like you did not “finish”	0.7 ± 0.9	0.6 ± 0.9	0.7 ± 0.9	0.9 ± 1.0	0.1152	0.8 ± 1.0	0.7 ± 0.9	0.7 ± 1.0	0.2363
bowel movements that were too hard	0.7 ± 1.0	0.6 ± 0.9	0.7 ± 1.0	0.8 ± 1.1	0.7807	0.8 ± 1.1	0.7 ± 1.0	0.6 ± 0.9	0.1420
bowel movements that were too small	0.5 ± 0.9	0.5 ± 0.9	0.5 ± 0.9	0.6 ± 1.0	0.5200	0.6 ± 0.9	0.5 ± 0.9	0.5 ± 0.8	0.0793
straining or squeezing to try to pass bowel movements	0.8 ± 1.1	0.7 ± 0.9	0.9 ± 1.1	0.9 ± 1.1	0.1599	1.0 ± 1.1	0.9 ± 1.1	0.6 ± 1.0	0.0017
feeling like you have to pass a bowel movement but you could not (false alarm)	0.5 ± 0.9	0.5 ± 0.9	0.5 ± 0.9	0.6 ± 1.0	0.4203	0.6 ± 1.0	0.5 ± 0.8	0.5 ± 0.9	0.2299
FACT-G7_sum of points ^#^	9.2 ± 5.3	7.7 ± 5.0	9.3 ± 4.8	10.7 ± 5.5	<0.0001	10.2 ± 5.3	9.3 ± 5.4	8.2 ± 4.9	0.0023
terciles									
bottom	35.5	47.3	33.9	25.1		28.4	35.9	42.1	
middle	30.5	28.3	31.7	31.7	0.0001	30.6	28.8	32.2	0.0203
upper	34.0	24.5	34.4	43.2		41.0	35.3	25.7	
FACT-G7_Components (points) ^#^									
I have a lack of energy	1.9 ± 1.2	1.7 ± 1.2	2.0 ± 1.1	2.1 ± 1.2	0.0057	2.1 ± 1.2	1.8 ± 1.2	1.8 ± 1.2	0.0192
I have pain	1.0 ± 1.1	0.7 ± 1.0	0.9 ± 1.0	1.3 ± 1.3	<0.0001	1.1 ± 1.1	0.9 ± 1.2	0.9 ± 1.1	0.0671
I have nausea	0.4 ± 0.8	0.3 ± 0.7	0.4 ± 0.7	0.5 ± 0.9	0.1735	0.4 ± 0.8	0.4 ± 0.9	0.3 ± 0.7	0.9127
I worry that my condition will get worse	1.1 ± 1.2	0.9 ± 1.0	1.2 ± 1.2	1.3 ± 1.3	0.0200	1.3 ± 1.2	1.2 ± 1.3	0.9 ± 1.1	0.0072
I am not sleeping well	1.7 ± 1.3	1.4 ± 1.3	1.7 ± 1.3	1.9 ± 1.4	0.0002	1.8 ± 1.3	1.6 ± 1.3	1.6 ± 1.4	0.1022
I am not able to enjoy life	1.5 ± 1.2	1.3 ± 1.1	1.5 ± 1.1	1.8 ± 1.3	0.0006	1.6 ± 1.3	1.6 ± 1.2	1.3 ± 1.1	0.0100
I am not content with the quality of my life right now	1.7 ± 1.2	1.5 ± 1.2	1.7 ± 1.2	1.9 ± 1.3	0.0078	1.9 ± 1.3	1.7 ± 1.2	1.4 ± 1.1	0.0028

%—sample percentage; ^#^ mean and standard deviation (SD); *p*-value—level of significance verified with chi^2^ test (categorical variables) or Kruskal–Wallis’ test (continuous variables); *p* < 0.05—statistically significant.

**Table 6:** Odds ratios (ORs (95% CI)) of the GSRS by the adherence to the dietary patterns among COVID-19 patients (*n* = 550).

Dietary Patterns	Terciles/ Levels	GSRS							
		Terciles							
		Middle (Ref. Bottom)				Upper (Ref. Bottom)			
		OR_crude_	95% CI	OR_adj_	95% CI	OR_crude_	95% CI	OR_adj_	95% CI
‘Processed high fat, sugar, salt, meat, dairy, and potatoes’	bottom (ref.)	1.00		1.00		1.00		1.00	
	middle	1.28	0.78; 2.11	1.23	0.72; 2.11	1.93 **	1.17; 3.20	2.21 **	1.28; 3.83
	upper	1.04	0.62; 1.73	1.40	0.78; 2.52	2.07 **	1.26; 3.40	3.12 ***	1.74; 5.63
	score (1-point increase)	1.00	0.79; 1.28	1.17	0.92; 1.49	1.26 *	1.03; 1.55	1.47 **	1.16; 1.86
‘Semi-vegetarian’	bottom (ref.)	1.00		1.00		1.00		1.00	
	middle	0.83	0.36; 1.91	0.75	0.42; 1.34	0.68	0.42; 1.10	0.57 *	0.33; 0.97
	upper	0.97	0.58; 1.61	0.85	0.48; 1.50	0.46 **	0.28; 0.75	0.31 ***	0.17; 0.56
	score (1-point increase)	0.98	0.80; 1.21	0.94	0.76; 1.18	0.70 ***	0.56; 0.86	0.62 ***	0.49; 0.79

ref.—reference category; OR_crude_—crude model; OR_adj_—model adjusted for age (years), gender (men, women), place of residence, educational level, ever smoking status (no, yes, occasionally), chronic diseases (no, yes), taking medication (no, yes), vitamin/mineral supplements use within the last 12 months (no, yes), special diet or intake restrictions (no, yes), and hospitalization (no, yes); 95% CI—95% confidence interval; *p*-value—the level of significance was assessed by Wald’s test; * *p* < 0.05, ** *p* < 0.01, *** *p* < 0.001.

**Table 7:** Odds ratios (ORs (95% CI)) of the PAC-SYM by the adherence to the dietary patterns among COVID-19 patients (*n* = 550).

Dietary Patterns	Terciles/ Levels	PAC-SYM							
		Terciles							
		Middle (Ref. Bottom)				Upper (Ref. Bottom)			
		OR_crude_	95% CI	OR_adj_	95% CI	OR_crude_	95% CI	OR_adj_	95% CI
‘Processed high fat, sugar, salt, meat, dairy, and potatoes’	bottom (ref.)	1.00		1.00		1.00		1.00	
	middle	0.98	0.58; 1.65	0.93	0.54; 1.61	1.56	0.96; 2.55	1.56	0.92; 2.64
	upper	1.08	0.64; 1.84	1.38	0.76; 2.54	2.19 **	1.35; 3.57	3.11 ***	1.76; 5.48
	score (1-point increase)	0.98	0.78; 1.23	1.05	0.81; 1.35	1.32 **	1.09; 1.60	1.50 ***	1.20; 1.87
‘Semi-vegetarian’	bottom (ref.)	1.00		1.00		1.00		1.00	
	middle	0.76	0.43; 1.31	0.66	0.37; 1.17	0.61 *	0.38; 0.98	0.52 *	0.31; 0.88
	upper	0.52 *	0.31; 0.90	0.47 *	0.26; 0.84	0.44 ***	0.27; 0.71	0.35 ***	0.20; 0.60
	score (1-point increase)	0.80 *	0.64; 0.99	0.78 *	0.61; 0.98	0.70 ***	0.57; 0.86	0.64 ***	0.51; 0.81

ref.—reference category; OR_crude_—crude model; OR_adj_—model adjusted for age (years), gender (men, women), place of residence, educational level, ever smoking status (no, yes, occasionally), chronic diseases (no, yes), taking medication (no, yes), vitamin/mineral supplements use within the last 12 months (no, yes), special diet or intake restrictions (no, yes), and hospitalization (no, yes); 95% CI—95% confidence interval; *p*-value—the level of significance was assessed by Wald’s test; * *p* < 0.05, ** *p* < 0.01, *** *p* < 0.001.

**Table 8:** Odds ratios (ORs (95% CI)) of the FACT-G7 by the adherence to the dietary patterns among COVID-19 patients (*n* = 550).

Dietary Patterns	Terciles/ Levels	FACT-G7							
		Terciles							
		Middle (Ref. Bottom)				Upper (Ref. Bottom)			
		OR_crude_	95% CI	OR_adj_	95% CI	OR_crude_	95% CI	OR_adj_	95% CI
‘Processed high fat, sugar, salt, meat, dairy, and potatoes’	bottom (ref.)	1.00		1.00		1.00		1.00	
	middle	1.57	0.95; 2.58	1.72 *	1.01; 2.95	1.96 **	1.19; 3.25	2.22 **	1.27; 3.89
	upper	2.11 **	1.25; 3.55	2.33 **	1.26; 4.32	3.32 ***	1.99; 5.55	4.55 ***	2.43; 8.50
	score (1-point increase)	1.28 *	1.04; 1.60	1.33 *	1.04; 1.71	1.51 ***	1.22; 1.87	1.54 ***	1.21; 1.97
‘Semi-vegetarian’	bottom (ref.)	1.00		1.00		1.00		1.00	
	middle	0.75	0.44; 1.26	0.74	0.42; 1.29	0.68	0.42; 1.12	0.70	0.41; 1.22
	upper	0.71	0.43; 1.19	0.80	0.46; 1.40	0.42 ***	0.25; 0.70	0.45 **	0.25; 0.78
	score (1-point increase)	0.88	0.72; 1.08	0.91	0.74; 1.14	0.68 ***	0.54; 0.85	0.70 **	0.55; 0.89

ref.—reference category; OR_crude_—crude model; OR_adj_—model adjusted for age (years), gender (men, women), place of residence, educational level, ever smoking status (no, yes, occasionally), chronic diseases (no, yes), taking medication (no, yes), vitamin/mineral supplements used within the last 12 months (no, yes), special diet or intake restrictions (no, yes), and hospitalization (no, yes); 95% CI—95% confidence interval; *p*-value—the level of significance was assessed by Wald’s test; * *p* < 0.05, ** *p* < 0.01, *** *p* < 0.001.

## Data Availability

All of the data are contained within the article and Supplementary Materials.

## Supplementary Materials

The following supporting information can be downloaded at: https://www.mdpi.com/article/10.3390/nu17050800/s1. Table S1: Description of 23 food groups aggregated: data based on the 62-item FFQ-6^®^; Table S2: The mean (95% CI) of the frequency of food consumption by dietary patterns among the COVID-19 patients (times/day); Table S3: The mean (SD) of the selected gastric scales by hospitalization among the COVID-19 patients; Table S4: The sample characteristics by dietary patterns among the COVID-19 patients (%) or mean (SD); Table S5: The mean (95% CI) of the frequency of food consumption by hospitalization among the COVID-19 patients; Table S6: The selected gastric scale components by dietary patterns among the COVID-19 patients (%).

## References

[1] WHO Coronavirus (COVID-19) Dashboard. https://covid19.who.int.

[2] Huang C., Wang Y., Li X., Ren L., Zhao J., Hu Y., Zhang L., Fan G., Xu J., Gu X. (2020). Clinical Features of Patients Infected with 2019 Novel Coronavirus in Wuhan, China. Lancet Lond. Engl..

[3] Xiao F., Tang M., Zheng X., Liu Y., Li X., Shan H. (2020). Evidence for Gastrointestinal Infection of SARS-CoV-2. Gastroenterology.

[4] Gu J., Han B., Wang J. (2020). COVID-19: Gastrointestinal Manifestations and Potential Fecal–Oral Transmission. Gastroenterology.

[5] Han C., Duan C., Zhang S., Spiegel B., Shi H., Wang W., Zhang L., Lin R., Liu J., Ding Z. (2020). Digestive Symptoms in COVID-19 Patients With Mild Disease Severity: Clinical Presentation, Stool Viral RNA Testing, and Outcomes. Am. J. Gastroenterol..

[6] Pan L., Mu M., Yang P., Sun Y., Wang R., Yan J., Li P., Hu B., Wang J., Hu C. (2020). Clinical Characteristics of COVID-19 Patients With Digestive Symptoms in Hubei, China: A Descriptive, Cross-Sectional, Multicenter Study. Am. J. Gastroenterol..

[7] Wang Y., Li Y., Zhang Y., Liu Y., Liu Y. (2022). Are Gastrointestinal Symptoms Associated with Higher Risk of Mortality in COVID-19 Patients? A Systematic Review and Meta-Analysis. BMC Gastroenterol..

[8] Lu R., Zhao X., Li J., Niu P., Yang B., Wu H., Wang W., Song H., Huang B., Zhu N. (2020). Genomic Characterisation and Epidemiology of 2019 Novel Coronavirus: Implications for Virus Origins and Receptor Binding. Lancet Lond. Engl..

[9] Yan R., Zhang Y., Li Y., Xia L., Guo Y., Zhou Q. (2020). Structural Basis for the Recognition of SARS-CoV-2 by Full-Length Human ACE2. Science.

[10] Kariyawasam J.C., Jayarajah U., Riza R., Abeysuriya V., Seneviratne S.L. (2021). Gastrointestinal Manifestations in COVID-19. Trans. R. Soc. Trop. Med. Hyg..

[11] Schloss J.V. (2023). Nutritional Deficiencies That May Predispose to Long COVID. Inflammopharmacology.

[12] Gombart A.F., Pierre A., Maggini S. (2020). A Review of Micronutrients and the Immune System–Working in Harmony to Reduce the Risk of Infection. Nutrients.

[13] Gracia Aznar A., Moreno Egea F., Gracia Banzo R., Gutierrez R., Rizo J.M., Rodriguez-Ledo P., Nerin I., Regidor P.-A. (2024). Pro-Resolving Inflammatory Effects of a Marine Oil Enriched in Specialized Pro-Resolving Mediators (SPMs) Supplement and Its Implication in Patients with Post-COVID Syndrome (PCS). Biomedicines.

[14] Balta M.G., Papathanasiou E., Christopoulos P.F. (2021). Specialized Pro-Resolving Mediators as Potential Regulators of Inflammatory Macrophage Responses in COVID-19. Front. Immunol..

[15] Cagnina R.E., Duvall M.G., Nijmeh J., Levy B.D. (2022). Specialized Pro-Resolving Mediators in Respiratory Diseases. Curr. Opin. Clin. Nutr. Metab. Care.

[16] Taha A.M., Shaarawy A.S., Omar M.M., Abouelmagd K., Shalma N.M., Alhashemi M., Ahmed H.M., Allam A.H., Abd-ElGawad M. (2022). Effect of Omega-3 Fatty Acids Supplementation on Serum Level of C-Reactive Protein in Patients with COVID-19: A Systematic Review and Meta-Analysis of Randomized Controlled Trials. J. Transl. Med..

[17] Sedighiyan M., Abdollahi H., Karimi E., Badeli M., Erfanian R., Raeesi S., Hashemi R., Vahabi Z., Asanjarani B., Mansouri F. (2021). Omega-3 Polyunsaturated Fatty Acids Supplementation Improve Clinical Symptoms in Patients with Covid-19: A Randomised Clinical Trial. Int. J. Clin. Pract..

[18] Toubal A., Kiaf B., Beaudoin L., Cagninacci L., Rhimi M., Fruchet B., da Silva J., Corbett A.J., Simoni Y., Lantz O. (2020). Mucosal-Associated Invariant T Cells Promote Inflammation and Intestinal Dysbiosis Leading to Metabolic Dysfunction during Obesity. Nat. Commun..

[19] Calder P.C., Carr A.C., Gombart A.F., Eggersdorfer M. (2020). Optimal Nutritional Status for a Well-Functioning Immune System Is an Important Factor to Protect against Viral Infections. Nutrients.

[20] García-Montero C., Fraile-Martínez O., Gómez-Lahoz A.M., Pekarek L., Castellanos A.J., Noguerales-Fraguas F., Coca S., Guijarro L.G., García-Honduvilla N., Asúnsolo A. (2021). Nutritional Components in Western Diet Versus Mediterranean Diet at the Gut Microbiota–Immune System Interplay. Implications for Health and Disease. Nutrients.

[21] Mardi A., Kamran A., Pourfarzi F., Zare M., Hajipour A., Doaei S., Abediasl N., Hackett D. (2023). Potential of Macronutrients and Probiotics to Boost Immunity in Patients with SARS-COV-2: A Narrative Review. Front. Nutr..

[22] Sidor A., Rzymski P. (2020). Dietary Choices and Habits during COVID-19 Lockdown: Experience from Poland. Nutrients.

[23] EUSurvey—Welcome. https://ec.europa.eu/eusurvey/home/welcome?language=en.

[24] Niedzwiedzka E., Wadolowska L., Kowalkowska J. (2019). Reproducibility of A Non-Quantitative Food Frequency Questionnaire (62-Item FFQ-6) and PCA-Driven Dietary Pattern Identification in 13–21-Year-Old Females. Nutrients.

[25] Armitage P., Berry G., Matthews J.N.S. (2013). Statistical Methods in Medical Research.

[26] Svedlund J., Sjödin I., Dotevall G. (1988). GSRS--a Clinical Rating Scale for Gastrointestinal Symptoms in Patients with Irritable Bowel Syndrome and Peptic Ulcer Disease. Dig. Dis. Sci..

[27] Kulich K.R., Madisch A., Pacini F., Piqué J.M., Regula J., Van Rensburg C.J., Újszászy L., Carlsson J., Halling K., Wiklund I.K. (2008). Reliability and Validity of the Gastrointestinal Symptom Rating Scale (GSRS) and Quality of Life in Reflux and Dyspepsia (QOLRAD) Questionnaire in Dyspepsia: A Six-Country Study. Health Qual. Life Outcomes.

[28] Yiannakou Y., Tack J., Piessevaux H., Dubois D., Quigley E.M.M., Ke M.Y., Silva S.D., Joseph A., Kerstens R. (2017). The PAC-SYM Questionnaire for Chronic Constipation: Defining the Minimal Important Difference. Aliment. Pharmacol. Ther..

[29] Frank L., Kleinman L., Farup C., Taylor L., Miner P. (1999). Psychometric Validation of a Constipation Symptom Assessment Questionnaire. Scand. J. Gastroenterol..

[30] Yanez B., Pearman T., Lis C.G., Beaumont J.L., Cella D. (2013). The FACT-G7: A Rapid Version of the Functional Assessment of Cancer Therapy-General (FACT-G) for Monitoring Symptoms and Concerns in Oncology Practice and Research. Ann. Oncol. Off. J. Eur. Soc. Med. Oncol..

[31] FACT-G7. https://www.facit.org/measures/fact-g7.

[32] Trujillo-Mayol I., Guerra-Valle M., Casas-Forero N., Sobral M.M.C., Viegas O., Alarcón-Enos J., Ferreira I.M.P.L.V.O., Pinho O. (2021). Western Dietary Pattern Antioxidant Intakes and Oxidative Stress: Importance During the SARS-CoV-2/COVID-19 Pandemic. Adv. Nutr..

[33] da Silva Oliveira E.K., Vieira T.d.S., de Souza O.F., Noll P.R.e.S., Bezerra I.M.P., Cavalcanti M.P.E., de Abreu L.C., Riera A.R.P. (2024). Consumption of Ultra-Processed Foods in the Brazilian Amazon during COVID-19. Nutrients.

[34] Malesza I.J., Malesza M., Walkowiak J., Mussin N., Walkowiak D., Aringazina R., Bartkowiak-Wieczorek J., Mądry E. (2021). High-Fat, Western-Style Diet, Systemic Inflammation, and Gut Microbiota: A Narrative Review. Cells.

[35] Clemente-Suárez V.J., Beltrán-Velasco A.I., Redondo-Flórez L., Martín-Rodríguez A., Tornero-Aguilera J.F. (2023). Global Impacts of Western Diet and Its Effects on Metabolism and Health: A Narrative Review. Nutrients.

[36] López-Taboada I., González-Pardo H., Conejo N.M. (2020). Western Diet: Implications for Brain Function and Behavior. Front. Psychol..

[37] Di Giosia P., Stamerra C.A., Giorgini P., Jamialahamdi T., Butler A.E., Sahebkar A. (2022). The Role of Nutrition in Inflammaging. Ageing Res. Rev..

[38] Mignogna C., Costanzo S., Ghulam A., Cerletti C., Donati M.B., de Gaetano G., Iacoviello L., Bonaccio M. (2021). Impact of Nationwide Lockdowns Resulting from the First Wave of the COVID-19 Pandemic on Food Intake, Eating Behaviors, and Diet Quality: A Systematic Review. Adv. Nutr..

[39] Bonaccio M., Costanzo S., Ruggiero E., Persichillo M., Esposito S., Olivieri M., Di Castelnuovo A., Cerletti C., Donati M.B., de Gaetano G. (2021). Changes in ultra-processed food consumption during the first Italian lockdown following the COVID-19 pandemic and major correlates: Results from two population-based cohorts. Public Health Nutr..

[40] Guasch-Ferré M., Willett W.C. (2021). The Mediterranean Diet and Health: A Comprehensive Overview. J. Intern. Med..

[41] Rahmati M., Fatemi R., Yon D.K., Won Lee S., Koyanagi A., Il Shin J., Smith L. (2022). The effect of adherence to high-quality dietary pattern on COVID-19 outcomes: A systematic review and meta-analysis. J. Med. Virol..

[42] Perez-Araluce R., Martinez-Gonzalez M.A., Fernandez-Lazaro C.I., Bes-Rastrollo M., Gea A., Carlos S. (2022). Mediterranean diet and the risk of COVID-19 in the ‘Seguimiento Universidad de Navarra’ cohort. Clin. Nutr..

[43] Tassakos A., Kloppman A., Chun J., Louie Y. (2025). The Impact of Diet Quality on COVID-19 Severity and Outcomes—A Scoping Review. Curr. Nutr. Rep..

[44] Chen J., Lu F., Shen B., Xu H., Chen Y., Hu Q., Xu A., Tung T.-H., Hong D. (2024). Associations between pre-infection serum vitamin D concentrations and Omicron COVID-19 incidence, severity and reoccurrence in elderly individuals. Public Health Nutr..

[45] Damayanthi H.D.W.T., Prabani K.I.P. (2021). Nutritional determinants and COVID-19 outcomes of older patients with COVID-19: A systematic review. Arch. Gerontol. Geriatr..

[46] Vogel-González M., Talló-Parra M., Herrera-Fernández V., Pérez-Vilaró G., Chillón M., Nogués X., Gómez-Zorrilla S., López-Montesinos I., Arnau-Barrés I., Sorli-Redó M.L. (2021). Low Zinc Levels at Admission Associates with Poor Clinical Outcomes in SARS-CoV-2 Infection. Nutrients.

[47] Barrett R., Youssef M., Shah I., Ioana J., Lawati A.A., Bukhari A., Hegarty S., Cormican L.J., Judge E., Burke C.M. (2022). Vitamin D Status and Mortality from SARS CoV-2: A Prospective Study of Unvaccinated Caucasian Adults. Nutrients.

[48] Younesian O., Khodabakhshi B., Abdolahi N., Norouzi A., Behnampour N., Hosseinzadeh S., Alarzi S.S.H., Joshaghani H. (2022). Decreased Serum Selenium Levels of COVID-19 Patients in Comparison with Healthy Individuals. Biol. Trace Elem. Res..

[49] Cioboata R., Vasile C.M., Bălteanu M.A., Georgescu D.E., Toma C., Dracea A.S., Nicolosu D. (2024). Evaluating Serum Calcium and Magnesium Levels as Predictive Biomarkers for Tuberculosis and COVID-19 Severity: A Romanian Prospective Study. Int. J. Mol. Sci..

[50] Taraszewska A. (2021). Risk Factors for Gastroesophageal Reflux Disease Symptoms Related to Lifestyle and Diet. Rocz. Panstw. Zakl. Hig..

[51] Lechien J.R., Bobin F., Mouawad F., Zelenik K., Calvo-Henriquez C., Chiesa-Estomba C.M., Enver N., Nacci A., Barillari M.R., Schindler A. (2019). Development of Scores Assessing the Refluxogenic Potential of Diet of Patients with Laryngopharyngeal Reflux. Eur. Arch. Otorhinolaryngol..

[52] Dağlı Ü., Kalkan İ.H. (2017). The Role of Lifestyle Changes in Gastroesophageal Reflux Diseases Treatment. Turk. J. Gastroenterol. Off. J. Turk. Soc. Gastroenterol..

[53] Kamiński M., Łoniewski I., Misera A., Marlicz W. (2019). Heartburn-Related Internet Searches and Trends of Interest across Six Western Countries: A Four-Year Retrospective Analysis Using Google Ads Keyword Planner. Int. J. Environ. Res. Public Health.

[54] Lacy B.E., Carter J., Weiss J.E., Crowell M.D. (2011). The Effects of Intraduodenal Nutrient Infusion on Serum CCK, LES Pressure, and Gastroesophageal Reflux. Neurogastroenterol. Motil..

[55] Baroni L., Bonetto C., Solinas I., Visaggi P., Galchenko A.V., Mariani L., Bottari A., Orazzini M., Guidi G., Lambiase C. (2023). Diets Including Animal Food Are Associated with Gastroesophageal Reflux Disease. Eur. J. Investig. Health Psychol. Educ..

[56] Zhang M., Hou Z.-K., Huang Z.-B., Chen X.-L., Liu F.-B. (2021). Dietary and Lifestyle Factors Related to Gastroesophageal Reflux Disease: A Systematic Review. Ther. Clin. Risk Manag..

[57] Mitsou E.K., Kakali A., Antonopoulou S., Mountzouris K.C., Yannakoulia M., Panagiotakos D.B., Kyriacou A. (2017). Adherence to the Mediterranean Diet Is Associated with the Gut Microbiota Pattern and Gastrointestinal Characteristics in an Adult Population. Br. J. Nutr..

[58] Barber C., Mego M., Sabater C., Vallejo F., Bendezu R.A., Masihy M., Guarner F., Espín J.C., Margolles A., Azpiroz F. (2021). Differential Effects of Western and Mediterranean-Type Diets on Gut Microbiota: A Metagenomics and Metabolomics Approach. Nutrients.

[59] Barberio B., Judge C., Savarino E.V., Ford A.C. (2021). Global Prevalence of Functional Constipation According to the Rome Criteria: A Systematic Review and Meta-Analysis. Lancet Gastroenterol. Hepatol..

[60] Nag A., Martin S.A., Mladsi D., Olayinka-Amao O., Purser M., Vekaria R.M. (2020). The Humanistic and Economic Burden of Chronic Idiopathic Constipation in the USA: A Systematic Literature Review. Clin. Exp. Gastroenterol..

[61] Vriesman M.H., Koppen I.J.N., Camilleri M., Di Lorenzo C., Benninga M.A. (2020). Management of Functional Constipation in Children and Adults. Nat. Rev. Gastroenterol. Hepatol..

[62] Lai H., Li Y., He Y., Chen F., Mi B., Li J., Xie J., Ma G., Yang J., Xu K. (2023). Effects of Dietary Fibers or Probiotics on Functional Constipation Symptoms and Roles of Gut Microbiota: A Double-Blinded Randomized Placebo Trial. Gut Microbes.

[63] Chey S.W., Chey W.D., Jackson K., Eswaran S. (2021). Exploratory Comparative Effectiveness Trial of Green Kiwifruit, Psyllium, or Prunes in US Patients with Chronic Constipation. Am. J. Gastroenterol..

[64] Khavandegar A., Heidarzadeh A., Angoorani P., Hasani-Ranjbar S., Ejtahed H.-S., Larijani B., Qorbani M. (2024). Adherence to the Mediterranean Diet Can Beneficially Affect the Gut Microbiota Composition: A Systematic Review. BMC Med. Genom..

[65] Duncanson K., Williams G., Hoedt E.C., Collins C.E., Keely S., Talley N.J. (2024). Diet-Microbiota Associations in Gastrointestinal Research: A Systematic Review. Gut Microbes.

[66] Chooi Y.C., Zhang Q.A., Magkos F., Ng M., Michael N., Wu X., Volchanskaya V.S.B., Lai X., Wanjaya E.R., Elejalde U. (2024). Effect of an Asian-Adapted Mediterranean Diet and Pentadecanoic Acid on Fatty Liver Disease: The TANGO Randomized Controlled Trial. Am. J. Clin. Nutr..

[67] Zhao Y., Zhan J., Wang Y., Wang D. (2022). The Relationship Between Plant-Based Diet and Risk of Digestive System Cancers: A Meta-Analysis Based on 3,059,009 Subjects. Front. Public Health.

[68] Losno E.A., Sieferle K., Perez-Cueto F.J.A., Ritz C. (2021). Vegan Diet and the Gut Microbiota Composition in Healthy Adults. Nutrients.

[69] Del Bo’ C., Bernardi S., Cherubini A., Porrini M., Gargari G., Hidalgo-Liberona N., González-Domínguez R., Zamora-Ros R., Peron G., Marino M. (2021). A Polyphenol-Rich Dietary Pattern Improves Intestinal Permeability, Evaluated as Serum Zonulin Levels, in Older Subjects: The MaPLE Randomised Controlled Trial. Clin. Nutr. Edinb. Scotl..

[70] Kim C.H. (2023). Complex Regulatory Effects of Gut Microbial Short-Chain Fatty Acids on Immune Tolerance and Autoimmunity. Cell. Mol. Immunol..

[71] Singh R.K., Chang H.-W., Yan D., Lee K.M., Ucmak D., Wong K., Abrouk M., Farahnik B., Nakamura M., Zhu T.H. (2017). Influence of Diet on the Gut Microbiome and Implications for Human Health. J. Transl. Med..

[72] Muscogiuri G., Pugliese G., Barrea L., Savastano S., Colao A. (2020). Commentary: Obesity: The “Achilles Heel” for COVID-19?. Metabolism.

[73] Ruiz-Roso M.B., Knott-Torcal C., Matilla-Escalante D.C., Garcimartín A., Sampedro-Nuñez M.A., Dávalos A., Marazuela M. (2020). COVID-19 Lockdown and Changes of the Dietary Pattern and Physical Activity Habits in a Cohort of Patients with Type 2 Diabetes Mellitus. Nutrients.

[74] Johnson A.N., Clockston R.L.M., Fremling L., Clark E., Lundeberg P., Mueller M., Graham D.J. (2023). Changes in Adults’ Eating Behaviors During the Initial Months of the COVID-19 Pandemic: A Narrative Review. J. Acad. Nutr. Diet..

[75] Rashidah N.H., Lim S.M., Neoh C.F., Majeed A.B.A., Tan M.P., Khor H.M., Tan A.H., Rehiman S.H., Ramasamy K. (2022). Differential Gut Microbiota and Intestinal Permeability between Frail and Healthy Older Adults: A Systematic Review. Ageing Res. Rev..

[76] Thompson F.E., Subar A.F. (2017). Assessment Methods for Research and Practice. Nutrition in the Prevention and Treatment of Disease. Dietary Assessment Methodology.

[77] FAO (2018). Dietary Assessment: A Resource Guide to Method Selection and Application in Low Resource Settings.

