# Supplementary material for: Associations Between Dietary Patterns and the Occurrence of Hospitalization and Gastrointestinal Disorders—A Retrospective Study of COVID-19 Patients
Source: Nutrients. 2025 Feb 26;17(5):800. doi: 10.3390/nu17050800 (PMC11901568; doi:10.3390/nu17050800)
Supplement: Supplementary file 1 [file nutrients-17-00800-s001.zip › nutrients-3483223-supplementary.pdf]

Table S1. Description of 22 food groups aggregated: data based on the 62-item FFQ-6® [Niedzwiedzka et al. 2019].

No	Food groups	Food groups description (food items)
1	Sugar, honey and sweets	Sugar added to beverages, such as tea, coffee, etc.;<br>Honey added to dishes and added to beverages;<br>Chocolates, chocolate sweets and chocolate bars, sugar confectionery (boiled sweets, hard caramels, jellied sweets, fudge, etc.), baked confectionery (biscuits, cream cakes, fruit cakes, sponge cakes, cheesecakes, doughnuts, poppy-seed cakes, muffins, croissants, etc.), ice-creams and custard.
2	Milk and milk beverages – natural and cheese curds	Milk and natural milk beverages (yoghurt, kefir, buttermilk), porridge, etc.<br>Cheese curd, natural cottage cheese, soft cheese, mozzarella, cottage cheese with herbs, etc.
3	Milk beverages – sweetened and flavoured cheese curds	Fruit yoghurts, yoghurts with chocolate flakes, flavoured buttermilk, hot chocolate, etc.<br>Flavoured curds (with fruit, chocolate, vanilla), etc.
4	Cheese	Hard cheese, blue cheese, processed cheese, cheese spreads, etc.
5	Eggs and egg dishes	Scrambled eggs, omelette, egg salad, cooked eggs.
6	Whole grains	Wholemeal wheat or rye bread, seeded loafs, pumpernickel, wholemeal cracker bread, etc.<br>Buckwheat groats, barley, brown rice, wholemeal pasta, etc.
7	Refined grains	Semolina, milled barley, pasta, white rice, rice flakes, etc.
8	Breakfast cereals	Prepared breakfast cereals, unboiled milk additives, e.g. muesli, cornflakes with or without additives
9	Animal fats	Butter;<br>Lard, pork fat, etc.<br>Cream (single, double, sour, used as an ingredient or added to beverages).
10	Other fats	all kinds of margarine in cubes (for frying, baking), margarine in cups (for spreading)<br>mayonnaise, salad dressings
11	Fruits	All kinds of fruits.
12	Vegetables	All kinds of vegetables (potatoes not included).
13	Legumes	Fresh and tinned legumes (corn, green peas, green beans, etc.);<br>Dry and processed pulses (beans (fava, butter kidney, broad, French, green), soya, peas, chickpea and processed pulses (baked beans, hummus, other bread spreads)).
14	Potatoes	Boiled, baked, French fries, potato rosti, gnocchi, etc.
15	Nuts and seeds	Peanuts, hazelnuts, walnuts, cashews, coconuts, chestnuts, peanut butter, chocolate-nut spread, etc.<br>Pumpkin seeds, sesame seeds, sunflower seeds, wheat germs, wheat bran, etc.
16	Processed meats	Sausages, bacon, reconstituted meat (sausages, meat loaf, hot-dogs, smoked sausages, bacon, etc.)<br>High quality cured meats (ham, poultry and pork-beef good quality cold meats, etc.)<br>Offal products (liver, blood sausage, sweetbread, liver pate, etc.).
17	White meat	Poultry and rabbit.
18	Fish	Lean fish (pollock, cod, perch, hake, carp to 1 kg, tuna, panga, trout, etc.)<br>Oily fish (salmon, sardines, herring, mackerel, eel, large carp, etc.).
19	Fruit, vegetable or vegetable-fruit juices	Fruit juices and nectars (mixed fruit juice, orange, grapefruit, apple, pear, grape, blackcurrant, cherry juice)<br>Vegetable and vegetable-fruit juices (mixed vegetable juice, tomato, carrot and carrot-fruit juice).
20	Sweetened beverages	All kinds of sweetened beverages.
21	Alcoholic drinks	All kinds of alcohol, including beer, wine, drinks, vodka, and spirits
22	Salty snacks	Chipsy, crackers, salted sticks, salted peanuts, etc.

Table S2. The mean (95%CI) of the frequency of food consumption (times/day) by dietary patterns (tertiles) among the COVID-19 patients.

Food groups#	Dietary Patterns (tertiles)					
	**'Processed high-in fat, sugar and salt foods, meats, dairy, and potatoes'**			**'Semi-vegetarian'**		
	bottom	middle	upper	bottom	middle	upper
Size (n)	184	183	183	183	184	183
Sugar, honey and sweets	0.4 (0.3, 0.4)	0.8 (0.7, 0.9)	1.5 (1.4, 1.7)	1.1 (1.0, 1.3)	0.8 (0.7, 1.0)	0.7 (0.6, 0.8)
Milk and milk beverages – natural and cheese curds	0.5 (0.4, 0.6)	0.8 (0.7, 0.9)	1.1 (1.0, 1.2)	0.5 (0.4, 0.5)	0.7 (0.6, 0.8)	1.2 (1.1, 1.3)
Milk beverages – sweetened and flavoured cheese curds	0.0 (0.0, 0.0)	0.1 (0.0, 0.1)	0.2 (0.1, 0.2)	0.1 (0.1, 0.1)	0.1 (0.1, 0.1)	0.1 (0.1, 0.1)
Cheese	0.1 (0.1, 0.1)	0.3 (0.3, 0.4)	0.6 (0.5, 0.6)	0.3 (0.2, 0.3)	0.3 (0.3, 0.4)	0.4 (0.3, 0.5)
Eggs and egg dishes	0.4 (0.4, 0.5)	0.5 (0.4, 0.5)	0.6 (0.5, 0.6)	0.3 (0.3, 0.4)	0.5 (0.4, 0.5)	0.7 (0.6, 0.8)
Whole grains	0.8 (0.7, 0.9)	0.9 (0.8, 1.0)	0.9 (0.8, 1.0)	0.4 (0.4, 0.5)	0.9 (0.8, 0.9)	1.3 (1.2, 1.4)
Refined grains	0.2 (0.1, 0.2)	0.4 (0.3, 0.5)	0.8 (0.8, 0.9)	0.5 (0.4, 0.6)	0.4 (0.3, 0.5)	0.5 (0.4, 0.6)
Breakfast cereals	0.0 (0.0, 0.0)	0.1 (0.0, 0.1)	0.1 (0.1, 0.1)	0.0 (0.0, 0.1)	0.0 (0.0, 0.1)	0.1 (0.1, 0.1)
Animal fats	0.4 (0.3, 0.4)	0.7 (0.6, 0.8)	1.2 (1.1, 1.3)	0.7 (0.6, 0.8)	0.8 (0.7, 0.9)	0.8 (0.7, 0.9)
Other fats	0.0 (0.0, 0.0)	0.1 (0.1, 0.1)	0.3 (0.2, 0.3)	0.1 (0.1, 0.2)	0.2 (0.1, 0.2)	0.1 (0.1, 0.2)
Fruits	0.8 (0.8, 0.9)	0.9 (0.8, 0.9)	0.8 (0.7, 0.9)	0.5 (0.5, 0.6)	0.8 (0.7, 0.8)	1.2 (1.1, 1.3)
Vegetables	1.3 (1.2, 1.3)	1.1 (1.0, 1.1)	1.1 (1.0, 1.2)	0.7 (0.6, 0.7)	1.1 (1.0, 1.2)	1.6 (1.5, 1.7)
Legumes	0.5 (0.4, 0.6)	0.4 (0.3, 0.5)	0.4 (0.3, 0.5)	0.2 (0.1, 0.2)	0.3 (0.3, 0.4)	0.7 (0.6, 0.9)
Potatoes	0.2 (0.2, 0.2)	0.3 (0.3, 0.3)	0.5 (0.5, 0.6)	0.3 (0.2, 0.3)	0.4 (0.3, 0.4)	0.4 (0.3, 0.5)
Nuts and seeds	0.9 (0.8, 1.0)	0.7 (0.6, 0.8)	0.6 (0.5, 0.7)	0.3 (0.2, 0.3)	0.7 (0.6, 0.8)	1.2 (1.1, 1.3)
Processed meats	0.1 (0.1, 0.2)	0.3 (0.3, 0.4)	0.7 (0.6, 0.8)	0.4 (0.3, 0.5)	0.4 (0.3, 0.5)	0.3 (0.3, 0.4)
White meat	0.2 (0.1, 0.2)	0.3 (0.2, 0.3)	0.4 (0.3, 0.4)	0.2 (0.1, 0.2)	0.3 (0.2, 0.3)	0.4 (0.3, 0.4)
Fish	0.2 (0.2, 0.3)	0.2 (0.2, 0.3)	0.3 (0.2, 0.3)	0.1 (0.1, 0.2)	0.2 (0.2, 0.3)	0.3 (0.3, 0.4)
Fruit, vegetable or vegetable-fruit juices	0.5	0.4	0.5	0.5	0.5	0.4

	(0.4, 0.5)	(0.3, 0.5)	(0.4, 0.6)	(0.4, 0.5)	(0.4, 0.6)	(0.3, 0.5)
Sweetened beverages	0.1<br>(0.1, 0.1)	0.1<br>(0.1, 0.1)	0.1<br>(0.0, 0.1)	0.1<br>(0.0, 0.1)	0.1<br>(0.1, 0.1)	0.1<br>(0.1, 0.1)
Alcoholic drinks	0.2<br>(0.1, 0.2)	0.3<br>(0.2, 0.3)	0.2<br>(0.1, 0.2)	0.2<br>(0.1, 0.2)	0.2<br>(0.2, 0.3)	0.2<br>(0.1, 0.3)
Salty snacks	0.0<br>(0.0, 0.1)	0.1<br>(0.1, 0.1)	0.1<br>(0.1, 0.2)	0.1<br>(0.1, 0.2)	0.1<br>(0.1, 0.1)	0.1<br>(0.0, 0.1)

the frequency consumption was expressed as a times/day after assigning the values for categories of frequency consumption as follows: ‘never or almost never’=0; ‘once a month or less’=0.025; ‘several times a month’=0.1; ‘several times a week’=0.571; ‘daily’=1; ‘several times a day’=2; 95%CI – 95% confidence interval.

Table S3. The mean (SD) of the selected gastric scales by the hospitalization among the COVID-19 patients.

Variables	Total sample	Hospitalization		*p*-Value
		no	yes	
Size (n)	550	468	82	
GSRS_sum of points	30.1±11.7	30.4±11.6	28.9±12.4	0.1032
tertiles				
bottom	37.3	35.3	48.8	
middle	28.7	29.9	22.0	0.0609
upper	34.0	34.8	29.3	
GSRS_Components				
pain or discomfort in your upper abdomen or the pit of your stomach	2.3±1.4	2.4±1.4	2.1±1.4	0.0160
heartburn	1.5±1.1	1.5±1.0	1.9±1.3	0.0014
acid reflux	1.6±1.1	1.5±1.1	1.7±1.4	0.5919
hunger pains	2.0±1.2	2.0±1.2	2.1±1.3	0.7599
nausea	1.5±1.1	1.5±1.1	1.5±1.1	0.2991
rumbling	2.2±1.3	2.3±1.2	1.9±1.3	0.0030
bloated stomach	2.7±1.6	2.8±1.6	2.0±1.3	<0.0001
breaking wind	3.0±1.5	3.1±1.5	2.6±1.5	0.0031
constipation	1.9±1.5	2.0±1.5	1.9±1.3	0.9615
diarrhoea	1.5±1.1	1.4±1.0	1.7±1.3	0.0675
hard stools	2.0±1.4	2.0±1.4	2.2±1.6	0.3954
loose stools	1.7±1.2	1.7±1.2	1.8±1.2	0.5441
urgent need to have a bowel movement	1.6±1.1	1.6±1.1	1.7±1.2	0.7061
sensation of not completely emptying the bowels	2.0±1.4	2.0±1.4	1.8±1.3	0.2905
belching	2.6±1.6	2.7±1.6	2.1±1.4	0.0004
PAC-SYM_sum of points	7.5±7.8	7.7±7.7	6.8±8.1	0.0905
tertiles				
bottom	42.5	41.5	48.8	
middle	23.8	24.8	18.3	0.3447
upper	33.6	33.8	32.9	
PAC-SYM_Components				
discomfort in your abdomen	0.8±0.9	0.8±0.9	0.6±0.9	0.0670
pain in your abdomen	0.5±0.8	0.6±0.8	0.5±0.9	0.1247
bloating in your abdomen	1.2±1.1	1.2±1.1	0.9±1.0	0.0059
stomach cramps	0.5±0.8	0.5±0.8	0.5±0.8	0.7876
painful bowel movements	0.4±0.9	0.4±0.9	0.5±0.9	0.3301
rectal burning during or after a bowel movement	0.5±0.9	0.5±0.9	0.6±0.9	0.2200
rectal bleeding during or after a bowel movement	0.3±0.8	0.3±0.8	0.3±0.7	0.7666
incomplete bowel movement, like you didn't "finish"	0.7±0.9	0.7±1.0	0.7±0.9	0.5090
bowel movements that were too hard	0.7±1.0	0.7±1.0	0.7±1.1	0.7924
bowel movements that were too small	0.5±0.9	0.5±0.9	0.5±0.9	0.8949
straining or squeezing to try to pass bowel movements	0.8±1.1	0.8±1.1	0.8±1.0	0.6948
feeling like you have to pass a bowel movement but you couldn't (false alarm)	0.5±0.9	0.6±1.0	0.3±0.6	0.0123
FACT-G7_sum of points	9.2±5.3	9.0±5.2	10.4±5.5	0.0333
tertiles				
bottom	35.5	36.8	28.0	
middle	30.5	30.6	30.5	0.2155
upper	34.0	32.7	41.5	
FACT-G7_Components				
I have a lack of energy	1.9±1.2	1.9±1.1	2.0±1.4	0.3763
I have pain	1.0±1.1	0.9±1.1	1.3±1.3	0.0190
I have nausea	0.4±0.8	0.4±0.8	0.4±0.8	0.8133
I worry that my condition will get worse	1.1±1.2	1.1±1.2	1.5±1.3	0.0038
I am not sleeping well	1.7±1.3	1.6±1.3	2.0±1.4	0.0210
I am not able to enjoy life	1.5±1.2	1.5±1.2	1.5±1.4	0.4366
I am not content with the quality of my life right now	1.7±1.2	1.7±1.2	1.8±1.4	0.6425

Table S4. The sample characteristics by dietary patterns among the COVID-19 patients (%) or mean (SD).

**Variable**	**Total sample**	**Dietary Patterns (tertiles/levels)**							
		**'Processed high-in fat, sugar and salt foods, meats, dairy, and potatoes' DP1**				**'Semi-vegetarian' DP2**			
		**bottom**	**middle**	**upper**	***p*-Value**	**bottom**	**middle**	**upper**	***p*-Value**
Sample size (n)	550	184	183	183		183	184	183	
Hospitalization (yes)	14.9	7.1	13.1	24.6	<0.0001	20.8	15.8	8.2	0.0031
Gender									
men	10.2	7.1	9.3	14.2	0.0687	14.2	11.4	4.9	0.0106
women	89.8	92.9	90.7	85.8		85.8	88.6	95.1	
Age (years[#])	41.2±11.4	40.4±9.4	41.3±11.4	41.8±13.2	0.8928	41.7±12.3	41.9±11.9	39.8±9.8	0.4208
18.0-29.9	14.2	12.5	14.8	15.3		14.8	14.1	13.7	
30.0-39.9	34.9	34.8	33.3	36.6		32.2	34.2	38.3	
40.0-49.9	31.8	38.6	30.1	26.8	0.0621	29.5	31.5	34.4	0.1636
50.0-59.9	10.5	10.3	12.6	8.7		14.8	8.2	8.7	
≥60.0	8.5	3.8	9.3	12.6		8.7	12.0	4.9	
Place of residence									
village	14.3	12.6	12.7	17.6		19.2	13.0	10.6	
town <50,000 inhabitants	16.7	18.1	18.2	13.7		16.5	11.4	22.3	
city (50,000-200,000 inhabitants)	28.4	23.1	26.0	36.3	0.0265	31.9	32.1	21.2	0.0019
city (>200,000 inhabitants)	40.6	46.2	43.1	32.4		32.4	43.5	45.8	
Educational level									
primary	1.1	0.0	1.6	1.6		2.2	1.1	0.0	
basic vocational	3.3	1.6	3.3	4.9		1.1	5.5	3.3	
secondary	18.6	14.8	20.2	20.9	0.1297	24.2	18.6	13.1	0.0095
higher	77.0	83.6	74.9	72.5		72.5	74.9	83.6	
Chronic diseases	46.4	49.2	40.4	49.7	0.1361	48.6	45.1	45.6	0.7613
Taking medication	41.3	44.0	38.3	41.5	0.5305	43.2	39.1	41.5	0.7316
Ever smoker									
no	48.5	44.8	50.0	50.8		45.1	45.9	54.6	
yes	33.2	38.3	28.9	32.2	0.3528	36.8	32.6	30.1	0.2528
occasionally	18.3	16.9	21.1	16.9		18.1	21.5	15.3	
Current smoker	8.2	9.8	7.7	7.1	0.6091	11.0	7.6	6.1	0.2187
Vitamin/mineral supplements use	90.4	91.3	90.7	89.1	0.7545	88.0	89.1	94.0	0.1177
vitamin supplements	43.4	39.1	44.6	46.7	0.3292	47.2	42.9	40.2	0.4084

folic acids supplements	36.2	36.5	33.7	38.3	0.6638	31.4	36.4	40.8	0.1870
iron supplements	32.1	33.0	31.6	31.8	0.9592	32.6	27.6	36.1	0.2264
zinc supplements	49.7	59.7	47.1	42.4	0.0036	50.3	50.3	48.6	0.9352
vitamin C supplements	64.0	63.8	68.0	60.1	0.3037	62.9	68.0	61.1	0.3743
Dietary habits									
Number of meals									
1-2	4.0	5.4	3.8	2.7		5.5	4.2	2.2	
3	33.5	37.0	29.5	33.9		37.7	29.3	33.3	
4	50.0	46.7	55.2	48.1	0.4265	45.4	54.3	50.3	0.4949
≥5	12.5	10.9	11.5	15.3		11.5	12.0	14.2	
Regular meal times									
no	17.8	10.9	18.6	24.0		26.2	13.6	13.7	
rather yes	60.9	63.0	63.4	56.3	0.0107	55.2	72.3	55.2	<0.0001
definitely yes	21.3	26.1	18.0	19.7		18.6	14.1	31.1	
Special diet or intake restrictions	58.5	67.9	63.4	44.3	<0.0001	48.6	62.0	65.0	0.0033
Overall decrease in food consumption	38.5	40.8	42.1	32.8	0.1418	33.9	41.8	39.9	0.2633
Restriction in consumption of:									
sugar and sweets	61.1	73.4	61.2	48.6	<0.0001	51.4	62.5	69.4	0.0017
potatoes and cereals	20.0	32.1	18.6	9.3	<0.0001	21.9	21.2	16.9	0.4426
fish	5.8	9.8	5.5	2.2	0.0077	6.6	4.9	6.0	0.7853
meat and meat products	33.6	49.5	36.1	15.3	<0.0001	23.0	37.5	40.4	0.0008
raw vegetables	1.8	1.6	2.2	1.6	0.9014	1.6	2.2	1.6	0.9066
fruits	6.7	9.2	5.5	5.5	0.2490	10.4	7.1	2.7	0.0137
dairy	31.1	44.6	30.6	18.0	<0.0001	32.2	34.2	26.8	0.2788
fats	20.5	26.6	21.3	13.7	0.0084	15.3	22.8	23.5	0.0979
foods in high fat content	36.4	55.4	34.4	19.1	<0.0001	31.1	31.0	47.0	0.0012

self-declared use of vitamin and/or mineral supplements within the last 12 months; %—sample percentage; #mean and standard deviation (SD); *p*-value—level of significance verified with chi^2 test (categorical variables) or Kruskal-Wallis' test (continuous variables); $p < 0.05$—statistically significant.

Table S5. The mean (95%CI) of the frequency of food consumption by the hospitalization among the COVID-19 patients.

Variables	Total sample	Hospitalization		*p*-Value
		no	yes	
Size (n)	550	468	82	
Frequency of food consumption (times/day)[&]				
Sugar, honey and sweets	0.9 (0.8, 1.0)	0.8 (0.7, 0.9)	1.3 (1.1, 1.6)	0.0001
Milk and milk beverages – natural and cheese curds	0.8 (0.7, 0.9)	0.8 (0.7, 0.9)	0.8 (0.7, 0.9)	0.1416
Milk beverages – sweetened and flavoured cheese curds	0.1 (0.1, 0.1)	0.1 (0.1, 0.1)	0.2 (0.1, 0.3)	<0.0001
Cheese	0.3 (0.3, 0.4)	0.3 (0.3, 0.4)	0.4 (0.3, 0.4)	0.3541
Eggs and egg dishes	0.5 (0.5, 0.5)	0.5 (0.5, 0.6)	0.4 (0.3, 0.4)	0.0025
Whole grains	0.9 (0.8, 0.9)	0.9 (0.8, 1.0)	0.7 (0.6, 0.9)	0.0281
Refined grains	0.5 (0.4, 0.5)	0.5 (0.4, 0.5)	0.5 (0.4, 0.6)	0.6650
Breakfast cereals	0.1 (0.0, 0.1)	0.1 (0.0, 0.1)	0.1 (0.0, 0.1)	0.2182
Animal fats	0.8 (0.7, 0.8)	0.7 (0.7, 0.8)	1.0 (0.9, 1.2)	0.0001
Other fats	0.1 (0.1, 0.2)	0.1 (0.1, 0.2)	0.2 (0.1, 0.3)	0.2506
Fruits	0.8 (0.8, 0.9)	0.8 (0.8, 0.9)	0.8 (0.7, 0.9)	0.3984
Vegetables	1.1 (1.1, 1.2)	1.2 (1.1, 1.2)	0.9 (0.8, 1.0)	0.0001
Legumes	0.4 (0.4, 0.5)	0.4 (0.4, 0.5)	0.3 (0.2, 0.4)	0.0056
Potatoes	0.3 (0.3, 0.4)	0.3 (0.3, 0.4)	0.5 (0.4, 0.5)	0.0005
Nuts and seeds	0.7 (0.7, 0.8)	0.8 (0.7, 0.8)	0.5 (0.4, 0.6)	0.0004
Processed meats	0.4 (0.3, 0.4)	0.3 (0.3, 0.4)	0.7 (0.6, 0.9)	<0.0001
White meat	0.3 (0.3, 0.3)	0.3 (0.2, 0.3)	0.3 (0.3, 0.4)	0.0213
Fish	0.2 (0.2, 0.3)	0.2 (0.2, 0.3)	0.3 (0.2, 0.3)	0.3837
Fruit, vegetable or vegetable-fruit juices	0.4 (0.4, 0.5)	0.4 (0.4, 0.5)	0.6 (0.5, 0.8)	0.0606
Sweetened beverages	0.1 (0.1, 0.1)	0.1 (0.1, 0.1)	0.1 (0.0, 0.1)	0.3500
Alcoholic drinks	0.2 (0.2, 0.2)	0.2 (0.2, 0.3)	0.0 (0.0, 0.1)	0.0017
Salty snacks	0.1 (0.1, 0.1)	0.1 (0.1, 0.1)	0.0 (0.0, 0.1)	<0.0001

[&]data from the 62-item FFQ-6®, the frequency consumption was expressed as a times/day after assigning the values for categories of frequency consumption as follows: 'never or almost never'=0; 'once a month or less'=0.025; 'several times a month'=0.1; 'several times a week'=0.571; 'daily'=1; 'several times a day'=2; $p < 0.05$—statistically significant; 95%CI – 95% confidence interval.

Table S6. The selected gastric scales components by dietary patterns among the COVID-19 patients (%).

Variable	Total sample	Dietary Patterns (tertiles/levels)							
		'Processed high-in fat, sugar and salt foods, meats, dairy, and potatoes'				'Semi-vegetarian'			
		**bottom**	**middle**	**upper**	***p*-Value**	**bottom**	**middle**	**upper**	***p*-Value**
Sample size (n)	550	184	183	183		183	184	183	
GSRS_Components									
Pain or discomfort in your upper abdomen or the pit of your stomach									
no discomfort	38.7	41.8	35.5	38.8		36.1	40.8	39.3	
slight discomfort	21.8	21.7	25.1	18.6		16.9	24.5	24.0	
mild discomfort	17.5	19.0	15.8	17.5		18.0	14.1	20.2	
moderate discomfort	13.1	12.0	13.1	14.2	0.5546	16.9	9.8	12.6	0.0611
moderately severe discomfort	6.5	4.9	6.6	8.2		9.8	7.1	2.7	
severe discomfort	2.2	0.5	3.3	2.7		2.2	3.3	1.1	
very severe discomfort	0.2	0.0	0.5	0.0		0.0	0.5	0.0	
Heartburn									
no discomfort	75.5	82.1	77.0	67.2		73.8	75.0	77.6	
slight discomfort	9.6	7.6	10.9	10.4		9.3	8.7	10.9	
mild discomfort	6.2	3.3	6.6	8.7		7.1	6.5	4.9	
moderate discomfort	5.6	4.3	3.8	8.7	0.0599	6.6	6.0	4.4	0.7393
moderately severe discomfort	2.4	1.6	1.6	3.8		1.6	3.3	2.2	
severe discomfort	0.7	1.1	0.0	1.1		1.6	0.5	0.0	
Acid reflux									
no discomfort	71.8	76.6	69.9	68.9		71.0	70.7	73.8	
slight discomfort	13.1	9.8	15.8	13.7		11.5	13.6	14.2	
mild discomfort	7.1	6.0	7.7	7.7		7.1	8.7	5.5	
moderate discomfort	4.4	5.4	3.8	3.8	0.5122	4.9	3.8	4.4	0.7116
moderately severe discomfort	1.8	1.1	1.6	2.7		2.2	1.1	2.2	
severe discomfort	1.5	1.1	1.1	2.2		2.7	1.6	0.0	
very severe discomfort	0.4	0.0	0.0	1.1		0.5	0.5	0.0	

Hunger pains									
no discomfort	46.5	59.2	44.3	36.1		40.4	46.2	53.0	
slight discomfort	25.3	25.0	28.4	22.4		25.1	27.2	23.5	
mild discomfort	13.6	9.8	12.0	19.1		16.9	10.9	13.1	
moderate discomfort	9.8	3.3	10.9	15.3	<0.0001	12.6	10.9	6.0	0.4030
moderately severe discomfort	4.0	2.7	4.4	4.9		3.8	4.3	3.8	
severe discomfort	0.4	0.0	0.0	1.1		0.5	0.0	0.5	
very severe discomfort	0.4	0.0	0.0	1.1		0.5	0.5	0.0	
Nausea									
no discomfort	75.8	78.3	74.9	74.3		73.8	73.9	79.8	
slight discomfort	10.2	9.8	9.3	11.5		10.4	12.0	8.2	
mild discomfort	6.0	4.3	7.7	6.0		5.5	6.5	6.0	
moderate discomfort	4.2	4.9	3.8	3.8	0.9103	5.5	3.8	3.3	0.8509
moderately severe discomfort	2.0	1.6	2.7	1.6		2.7	2.2	1.1	
severe discomfort	1.6	1.1	1.6	2.2		2.2	1.1	1.6	
very severe discomfort	0.2	0.0	0.0	0.5		0.0	0.5	0.0	
Rumbling									
no discomfort	37.6	45.1	31.7	36.1		36.1	37.5	39.3	
slight discomfort	27.6	26.1	31.1	25.7		25.1	28.3	29.5	
mild discomfort	17.5	16.3	18.0	18.0		18.6	15.8	18.0	
moderate discomfort	12.4	9.8	13.7	13.7	0.4431	14.8	12.0	10.4	0.5878
moderately severe discomfort	3.6	2.7	3.8	4.4		4.9	4.3	1.6	
severe discomfort	0.9	0.0	1.1	1.6		0.5	1.1	1.1	
very severe discomfort	0.4	0.0	0.5	0.5		0.0	1.1	0.0	
Bloated stomach									
no discomfort	28.9	32.1	21.9	32.8		25.1	34.8	26.8	
slight discomfort	27.6	32.1	29.5	21.3		24.6	25.5	32.8	
mild discomfort	13.5	12.0	18.6	9.8		13.1	10.3	16.9	
moderate discomfort	15.6	15.2	13.1	18.6	0.0055	19.7	14.7	12.6	0.2034
moderately severe discomfort	7.5	4.9	6.6	10.9		8.7	7.1	6.6	

severe discomfort	5.8	2.7	9.3	5.5		7.1	6.5	3.8	
very severe discomfort	1.1	1.1	1.1	1.1		1.6	1.1	0.5	
Breaking wind									
no discomfort	15.8	17.9	13.7	15.8		13.7	18.5	15.3	
slight discomfort	30.0	35.3	30.1	24.6		26.8	28.3	35.0	
mild discomfort	18.7	19.0	17.5	19.7		19.7	13.6	23.0	
moderate discomfort	20.4	20.1	19.7	21.3	0.1721	21.9	22.3	16.9	0.0739
moderately severe discomfort	8.0	3.8	9.8	10.4		8.2	10.9	4.9	
severe discomfort	5.1	2.2	6.6	6.6		8.2	4.3	2.7	
very severe discomfort	2.0	1.6	2.7	1.6		1.6	2.2	2.2	
Constipation									
no discomfort	60.2	66.8	57.4	56.3		55.2	58.7	66.7	
slight discomfort	14.9	13.6	15.3	15.8		15.8	14.1	14.8	
mild discomfort	8.9	7.1	9.3	10.4		10.4	8.2	8.2	
moderate discomfort	7.5	6.0	9.3	7.1	0.7965	8.2	7.6	6.6	0.0093
moderately severe discomfort	4.2	2.7	3.8	6.0		2.7	8.7	1.1	
severe discomfort	3.1	2.7	3.3	3.3		6.0	2.2	1.1	
very severe discomfort	1.3	1.1	1.6	1.1		1.6	0.5	1.6	
Diarrhoea									
no discomfort	76.9	81.0	74.3	75.4		76.5	74.5	79.8	
slight discomfort	10.5	10.9	10.4	10.4		13.1	9.8	8.7	
mild discomfort	4.7	3.3	7.7	3.3		2.7	6.0	5.5	
moderate discomfort	5.3	2.7	3.8	9.3	0.0563	3.8	7.1	4.9	0.3061
moderately severe discomfort	1.3	1.1	2.2	0.5		1.6	2.2	0.0	
severe discomfort	0.9	1.1	0.5	1.1		1.6	0.0	1.1	
very severe discomfort	0.4	0.0	1.1	0.0		0.5	0.5	0.0	
Hard stools									
no discomfort	54.2	59.2	50.8	52.5		47.5	56.0	59.0	
slight discomfort	20.9	23.9	24.0	14.8		25.7	14.7	22.4	
mild discomfort	9.1	8.2	8.2	10.9		7.7	9.8	9.8	

moderate discomfort	8.2	5.4	7.7	11.5	0.0240	7.7	13.0	3.8	0.0062
moderately severe discomfort	3.3	0.5	5.5	3.8		4.9	3.3	1.6	
severe discomfort	2.9	1.1	2.7	4.9		3.8	3.3	1.6	
very severe discomfort	1.5	1.6	1.1	1.6		2.7	0.0	1.6	
Loose stools									
no discomfort	62.5	69.0	59.0	59.6		61.7	63.6	62.3	
slight discomfort	19.6	15.2	23.5	20.2		21.3	16.3	21.3	
mild discomfort	7.3	8.2	5.5	8.2		5.5	8.2	8.2	
moderate discomfort	6.9	6.0	6.6	8.2	0.2650	6.6	8.2	6.0	0.5898
moderately severe discomfort	2.2	0.5	2.7	3.3		3.8	2.2	0.5	
severe discomfort	1.3	1.1	2.2	0.5		1.1	1.6	1.1	
very severe discomfort	0.2	0.0	0.5	0.0		0.0	0.0	0.5	
Urgent need to have a bowel movement									
no discomfort	67.5	72.8	65.0	64.5		63.4	63.6	75.4	
slight discomfort	17.3	15.8	19.1	16.9		21.9	17.4	12.6	
mild discomfort	5.8	6.0	6.0	5.5		5.5	7.6	4.4	
moderate discomfort	5.8	3.3	5.5	8.7	0.3145	6.0	6.5	4.9	0.4336
moderately severe discomfort	2.7	1.6	2.7	3.8		2.7	3.3	2.2	
severe discomfort	0.7	0.0	1.6	0.5		0.5	1.1	0.5	
very severe discomfort	0.2	0.5	0.0	0.0		0.0	0.5	0.0	
Sensation of not completely emptying the bowels									
no discomfort	53.5	57.1	53.0	50.3		49.7	55.4	55.2	
slight discomfort	21.1	21.7	23.0	18.6		20.2	20.1	23.0	
mild discomfort	10.9	12.0	8.7	12.0		15.3	8.7	8.7	
moderate discomfort	7.5	3.8	6.0	12.6	0.0576	4.9	10.3	7.1	0.1940
moderately severe discomfort	4.4	2.7	5.5	4.9		6.6	3.8	2.7	
severe discomfort	1.6	1.1	3.3	0.5		1.6	1.6	1.6	
very severe discomfort	1.1	1.6	0.5	1.1		1.6	0.0	1.6	
Belching									

no discomfort	31.6	34.8	26.2	33.9		29.0	34.2	31.7	
slight discomfort	27.3	31.0	31.7	19.1		26.2	25.5	30.1	
mild discomfort	14.0	13.0	14.8	14.2		15.3	10.9	15.8	
moderate discomfort	13.8	11.4	11.5	18.6	0.0863	10.9	16.8	13.7	0.3015
moderately severe discomfort	6.9	4.3	7.7	8.7		9.3	7.6	3.8	
severe discomfort	4.0	2.7	5.5	3.8		5.5	3.3	3.3	
very severe discomfort	2.4	2.7	2.7	1.6		3.8	1.6	1.6	
PAC-SYM_Components									
Discomfort in your abdomen									
did not occur	50.2	56.5	46.4	47.5		43.7	54.3	52.5	
mild	29.5	28.8	30.6	29.0		31.1	27.2	30.1	
moderate	14.0	10.3	16.4	15.3	0.3515	16.4	10.9	14.8	0.0825
severe	5.8	4.3	5.5	7.7		8.7	6.5	2.2	
very severe	0.5	0.0	1.1	0.5		0.0	1.1	0.5	
Pain in your abdomen									
did not occur	63.6	68.5	65.6	56.8		57.9	65.8	67.2	
mild	22.4	22.3	20.8	24.0		26.8	20.1	20.2	
moderate	10.7	8.2	9.8	14.2	0.0976	10.9	12.0	9.3	0.3448
severe	2.5	1.1	2.2	4.4		2.7	1.6	3.3	
very severe	0.7	0.0	1.6	0.5		1.6	0.5	0.0	
Bloating in your abdomen									
did not occur	31.1	33.7	26.2	33.3		27.3	35.9	30.1	
mild	35.3	40.2	38.3	27.3		36.1	29.9	39.9	
moderate	21.3	15.8	20.8	27.3	0.0710	21.3	24.5	18.0	0.3296
severe	10.0	8.2	12.0	9.8		12.0	8.2	9.8	
very severe	2.4	2.2	2.7	2.2		3.3	1.6	2.2	
Stomach cramps									
did not occur	71.5	79.9	71.6	62.8		67.8	70.7	76.0	
mild	15.5	12.0	15.8	18.6		19.7	13.6	13.1	
moderate	9.6	6.5	8.7	13.7	0.0636	8.2	13.0	7.7	0.3314

severe	2.7	1.1	3.3	3.8		3.8	2.2	2.2	
very severe	0.7	0.5	0.5	1.1		0.5	0.5	1.1	
Painful bowel movements									
did not occur	74.5	80.4	75.4	67.8		67.2	73.9	82.5	
mild	12.9	11.4	11.5	15.8		16.4	13.6	8.7	
moderate	8.5	5.4	8.7	11.5	0.3025	12.0	8.7	4.9	0.0071
severe	2.2	1.1	2.7	2.7		1.6	3.8	1.1	
very severe	1.8	1.6	1.6	2.2		2.7	0.0	2.7	
Rectal burning during or after a bowel movement									
did not occur	69.5	76.6	69.9	61.7		63.9	71.2	73.2	
mild	16.7	14.7	18.6	16.9		21.3	13.6	15.3	
moderate	10.0	6.5	8.7	14.8	0.0287	12.0	11.4	6.6	0.0739
severe	2.2	1.1	2.2	3.3		1.6	3.3	1.6	
very severe	1.6	1.1	0.5	3.3		1.1	0.5	3.3	
Rectal bleeding during or after a bowel movement									
did not occur	83.3	86.4	84.2	79.2		78.1	84.8	86.9	
mild	6.9	6.0	8.2	6.6		8.2	7.6	4.9	
moderate	6.0	3.3	4.4	10.4	0.1216	9.3	4.9	3.8	0.2255
severe	2.9	3.8	2.7	2.2		2.7	2.7	3.3	
very severe	0.9	0.5	0.5	1.6		1.6	0.0	1.1	
Incomplete bowel movement, like you didn't "finish"									
did not occur	52.2	54.9	53.0	48.6		47.0	54.3	55.2	
mild	29.3	33.2	28.4	26.2		32.2	27.2	28.4	
moderate	12.9	8.2	13.7	16.9	0.0494	14.2	14.1	10.4	0.4570
severe	4.2	1.6	3.8	7.1		4.4	4.3	3.8	
very severe	1.5	2.2	1.1	1.1		2.2	0.0	2.2	
Bowel movements that were too hard									
did not occur	60.7	60.9	59.0	62.3		56.8	59.8	65.6	
mild	19.3	23.9	20.8	13.1		21.3	16.8	19.7	

moderate	12.4	10.3	14.2	12.6	0.0560	13.7	14.1	9.3	0.3681
severe	5.8	3.3	4.4	9.8		5.5	8.2	3.8	
very severe	1.8	1.6	1.6	2.2		2.7	1.1	1.6	
Bowel movements that were too small									
did not occur	66.9	67.4	68.9	64.5		60.7	69.6	70.5	
mild	18.2	20.1	16.9	17.5		20.2	16.8	17.5	
moderate	9.8	8.2	10.9	10.4	0.4423	14.2	7.6	7.7	0.3979
severe	4.2	3.3	2.2	7.1		3.8	4.9	3.8	
very severe	0.9	1.1	1.1	0.5		1.1	1.1	0.5	
Straining or squeezing to try to pass bowel movements									
did not occur	52.0	55.4	51.4	49.2		44.8	47.8	63.4	
mild	24.0	27.7	20.2	24.0		27.9	25.5	18.6	
moderate	14.2	9.8	18.6	14.2	0.2016	16.9	15.8	9.8	0.0386
severe	8.0	6.0	8.2	9.8		7.7	9.2	7.1	
very severe	1.8	1.1	1.6	2.7		2.7	1.6	1.1	
Feeling like you have to pass a bowel movement but you couldn’t (false alarm)									
did not occur	68.2	70.1	68.3	66.1		64.5	66.8	73.2	
mild	16.7	18.5	19.1	12.6		18.0	19.6	12.6	
moderate	9.5	7.6	6.6	14.2	0.0766	10.9	9.2	8.2	0.1348
severe	4.5	2.2	5.5	6.0		3.8	4.3	5.5	
very severe	1.1	1.6	0.5	1.1		2.7	0.0	0.5	
FACT-G7_Components									
I have a lack of energy									
I completely disagree	13.8	18.5	11.5	11.5		9.8	15.8	15.8	
a little	20.4	24.5	17.5	19.1		17.5	20.7	23.0	
sometimes	36.5	34.2	41.5	33.9	0.0852	38.3	34.8	36.6	0.2572
quite often	18.9	14.1	19.7	23.0		20.2	20.7	15.8	
I totally agree	10.4	8.7	9.8	12.6		14.2	8.2	8.7	
I have pain									

I completely disagree	47.6	58.7	45.4	38.8		38.8	52.2	51.9	
a little	22.7	20.1	28.4	19.7		29.5	17.4	21.3	
sometimes	19.8	14.7	20.8	24.0	<0.0001	20.2	20.1	19.1	0.0618
quite often	6.4	4.9	3.8	10.4		8.7	5.4	4.9	
I totally agree	3.5	1.6	1.6	7.1		2.7	4.9	2.7	
I have nausea									
I completely disagree	74.7	78.8	74.9	70.5		73.8	75.0	75.4	
a little	15.3	13.6	14.8	17.5		15.3	15.2	15.3	
sometimes	7.3	4.9	8.2	8.7	0.1024	8.7	4.9	8.2	0.4294
quite often	2.0	2.7	2.2	1.1		1.6	3.3	1.1	
I totally agree	0.7	0.0	0.0	2.2		0.5	1.6	0.0	
I worry that my condition will get worse									
I completely disagree	39.6	46.2	37.7	35.0		30.6	41.8	46.4	
a little	28.0	26.6	29.0	28.4		32.8	22.8	28.4	
sometimes	18.2	17.9	19.1	17.5	0.0495	21.9	16.3	16.4	0.0131
quite often	8.9	8.2	8.7	9.8		8.7	13.0	4.9	
I totally agree	5.3	1.1	5.5	9.3		6.0	6.0	3.8	
I am not sleeping well									
I completely disagree	12.4	9.8	10.4	16.9		13.1	12.0	12.0	
a little	16.4	12.0	15.8	21.3		18.0	15.2	15.8	
sometimes	20.0	15.8	23.0	21.3	0.0018	24.6	20.1	15.3	0.3911
quite often	26.7	29.9	30.6	19.7		25.1	26.1	29.0	
I totally agree	24.5	32.6	20.2	20.8		19.1	26.6	27.9	
I am not able to enjoy life									
I completely disagree	7.3	4.9	7.1	9.8		9.3	7.1	5.5	
a little	14.5	8.7	9.8	25.1		16.9	14.7	12.0	
sometimes	22.9	23.9	27.3	17.5	<0.0001	26.2	27.2	15.3	0.0016
quite often	31.6	32.6	35.0	27.3		21.3	33.2	40.4	
I totally agree	23.6	29.9	20.8	20.2		26.2	17.9	26.8	
I am not content with the quality of my life									

right now									
I completely disagree	9.6	6.5	9.3	13.1		15.3	8.7	4.9	
a little	16.0	12.0	15.8	20.2		16.4	17.9	13.7	
sometimes	25.8	27.2	25.7	24.6	0.0135	26.2	27.2	24.0	0.0319
quite often	30.5	30.4	36.6	24.6		26.2	29.9	35.5	
I totally agree	18.0	23.9	12.6	17.5		15.8	16.3	21.9	

self-declared use of vitamin and/or mineral supplements within the last 12 months; %—sample percentage; #mean and standard deviation (SD); *p*-value—level of significance verified with chi^2 test (categorical variables) or Kruskal-Wallis' test (continuous variables); $p < 0.05$—statistically significant.
